# Dietary intake, quality, and assessment tools in individuals with problematic alcohol use: a scoping review and meta-analysis

**DOI:** 10.1038/s41398-026-03842-9

**Published:** 2026-01-28

**Authors:** Jennifer J. Barb, Lillian C. King, Shubhi Nanda, Donna Barnett, Valerie L. Darcey, Shanna Yang, Sara Turner, Ayaan Ahmed, Katherine A. Maki, Carlotta Vizioli, Gisela Butera, Mehdi Farokhnia, Gwenyth R. Wallen, Lorenzo Leggio

**Affiliations:** 1 Translational Biobehavioral and Health Promotion Branch, National Institutes of Health Clinical Center, Bethesda, MD US; 2 Nutrition Department, National Institutes of Health Clinical Center, Bethesda, MD US; 3 Nutritional and Metabolic Neuroimaging, Diabetes, Endocrinology and Obesity Branch, National Institute of Diabetes and Digestive and Kidney Diseases, Bethesda, MD US; 4 Clinical Psychoneuroendocrinology and Neuropsychopharmacology Section, Translational Addiction Medicine Branch, National Institute on Drug Abuse Intramural Research Program and National Institute on Alcohol Abuse, National Institutes of Health, Baltimore, MD US; 5 Division of Library Services, National Institutes of Health, Bethesda, MD US

**Keywords:** Scientific community, Addiction

## Abstract

Alcohol Use Disorder (AUD) is commonly associated with malnutrition, yet the relative contributions of inadequate intake versus alcohol-related metabolic disruption remain unclear. This scoping review summarizes existing literature on dietary intake patterns and diet quality among individuals with AUD, following the Preferred Reporting Items for Systematic reviews and Meta-Analyses extension for Scoping Reviews guidelines. A comprehensive, systematic search was conducted without date restrictions, and dietary intake was categorized by drinking status (active vs. abstinent). Across 41 included studies, only four reported on diet quality or assessed adherence to recommended nutrient intake. There was considerable variability in both the reporting of dietary variables and the assessment tools utilized across studies. Weighted averages showed that individuals with AUD generally had Body Mass Index (BMI) values in the normal range and reported adequate total caloric intake and macronutrient distribution during both active drinking and abstinence. However, despite seemingly sufficient intake, nutrient deficiencies are common in this population, likely due to alcohol-related interference with nutrient absorption, metabolism, and utilization. These findings underscore the need for AUD-specific nutritional guidelines, standardized dietary assessment methods, and more robust evaluations of diet quality. Integrating nutrition science into AUD research and clinical care may provide an opportunity to improve both treatment outcomes and long-term recovery.

## Introduction

Alcohol Use Disorder (AUD) is characterized by patterns of problematic alcohol consumption with detrimental social, occupational, and health consequences [1, 2]. It is a chronic and highly prevalent condition in the United States and worldwide [1, 3]. While the physiological, behavioral, and psychosocial aspects of AUD are well-documented, its intersection with nutrition remains underexplored despite growing evidence that nutritional status plays a critical role in disease progression, treatment response, and recovery outcomes [4].

Nutrition is a fundamental determinant of health and has unique relevance for individuals with AUD. Excessive alcohol consumption is often accompanied by reduced intake of nutrient-dense foods (primary malnutrition), impaired nutrient absorption, altered metabolic pathways, and increased nutrient excretion (secondary malnutrition), all of which contribute to heightened risk for adverse impacts on total health as malnutrition of any etiology can cause significant harm [4]. Research suggests that disruption of metabolic stability may also lead to enhanced dependence on alcohol in the AUD population provided that a lack of nutrients and hunger may intensify alcohol cravings [4, 5].

Diet quality, which can be optimized through a variety of nutrient dense, minimally processed foods that are low in added sugars, saturated fats and sodium, is influenced by several interrelated behavioral and physiological factors in individuals with AUD [6]. Aside from disrupting appetite regulation, chronic problematic alcohol use contributes to primary nutritional insufficiency via increasing intake of energy dense, nutrient poor foods during and between drinking episodes [7]. Chronic problematic alcohol use also impairs nutrient absorption in the gastrointestinal tract, and alters the metabolism of essential vitamins and minerals, particularly water soluble vitamins B1 (thiamine), B6 (pyridoxine), B12 (cyanocobalamin) and B9 (folate), and fat soluble vitamins like A and D, as well as electrolytes like magnesium and zinc, contributing to secondary malnutrition [7–9]. Alcohol’s diuretic effect further increases urinary excretion of B vitamins, magnesium, and zinc. B1 deficiency can contribute to heart failure and sudden death; furthermore, B1 deficiency can lead to Wernicke’s encephalopathy (WE) which can be reversed with supplementation, however, the chronic progression of this, Wernicke–Korsakoff syndrome, can become irreversible [10–12]. Disruption of absorption and metabolic processing leads to nutrient deficiencies but may also compound neurocognitive impairments, weaken immune function, and increase vulnerability to comorbid conditions such as liver, cardiovascular and metabolic diseases, and depression. For example, inadequate B vitamins compromise energy metabolism and neurotransmitter synthesis [13, 14], while magnesium and zinc depletion reduce inhibition of NMDA receptors, increasing risk of glutamate excitotoxicity that contributes to withdrawal severity and relapse [15]. Importantly, improvements in diet quality could have the potential to yield meaningful health benefits for this population particularly in those with other medical comorbidities that can be addressed with medical nutrition therapy, (e.g., alcohol-associated liver disease) [16]. However, for patients with AUD, we lack both a robust, consistent framework for dietary assessment and intervention strategies for improving nutrient status and dietary quality.

Historically, nutritional assessment of individuals with AUD has relied on indirect outcomes such as anthropometric data, serum biomarkers, or clinical symptoms related to nutrient deficiencies. However, general population norms may fail to accurately reflect the health of individuals with AUD. For example, preference for energy dense, nutrient poor foods can lead to higher body mass index (BMI) but suboptimal nutritional status. Indeed, the majority (88%) of patients at one alcohol treatment facility had a normal BMI, yet 53% were found to be at moderate or high risk of malnutrition when assessed by a medical professional using the Malnutrition Universal Screening Tool [17]. These findings highlight the limitations of conventional body composition measures in capturing the complex underpinnings of the nutritional status of people with AUD.

Dietary intake assessment tools, such as food frequency questionnaires (FFQs), 24 h dietary recalls, diet history interviews and food diaries, are commonly used in nutrition research. However, their implementation in clinical practice is challenged by limited staffing or other resources. Furthermore, these instruments may be subject to recall bias, a limitation reported across different patient populations, including those with AUD [8, 18]. Regardless of whether a tool has been specifically validated for individuals with AUD, all diet assessment tools face unique challenges. The lack of methodological consistency in how intake data are collected and reported not only limits cross-study comparability but also contributes to an incomplete understanding of dietary behaviors and nutritional risk in this high-need group.

Despite the importance of the nutritional status and dietary behaviors in people with AUD, this area of research has not been investigated in a systematic way. This scoping review and meta-analysis focuses specifically on dietary intake patterns and diet quality assessment. We synthesize the existing literature on dietary intake patterns and diet quality in individuals with AUD, with a secondary aim to catalog the diet assessment tools used in this population. Through this effort, we aim to describe dietary patterns and assessment tools in AUD-related clinical research and practice, identify methodological limitations, and highlight opportunities for future developments and future research related to malnutrition in the AUD population.

## Materials and methods

This scoping review was conducted following the five stage methodology first described by Arksey and O’Malley and updated by Joanna Briggs Institute, including: identification of the research question, identification of relevant studies, study selection, extraction and presentation of the data and data collation (Table 1) [19, 20]. Three databases were searched: PubMed/MEDLINE, Scopus and Web of Science using a combination of Medical Subject Headings (MeSH) terms and keywords procedure. Two searches were conducted: one in October 2023 with no date limitations and an update in July 2024. Four authors performed abstract screening to generate the list of full text review, and nine authors performed full text review and data extraction for all included studies. Covidence was used for abstract screening and full text review [21]. The following steps were followed as part of this scoping review: 1) Research question identification, 2) Identification of all studies with the search strategy and database searching, 3) Study screening from abstract to full-text review, 4) Extraction of relevant data and 5) Collating and dissemination of the results. This review is aimed at addressing the following three questions: *What are the typical dietary intake patterns of individuals with AUD during active drinking and abstinence (e.g., macro and micronutrient intake and dietary quality)*, *What types of diet quality metrics are reported for individuals with AUD* and *What tools are used to assess dietary intake in individuals diagnosed with AUD*. All methods in this scoping review were performed in accordance with the relevant guidelines and regulations. Informed consent was not needed for this study since this was a scoping review of published literature and published content would have included informed consent from study subjects. No images were published involving human research participants. For a full description of all methods, search terms and queries used for this scoping review, see Supplemental Material and Methods.

**Table 1:** Included Study Population Characteristics.

Metric	# of studies(%^a^ of total N = 41)	Sample Size (% of total N = 2727)	Weighted Average^b^
*Age*	38 (93%)	2581 (94.6%)	45.58
Missing Age	3 (7%)	146 (5.4%)	N/A
*Sex*	36 (88%)	Male 2064 (75.7%) Female 354 (13.0%)	N/A
Missing Sex	5 (12%)	309 (11.3%)	N/A
*Anthropometrics*			
Body Mass index (BMI) (kg/m^2^)	19 (46%)	1115 (40.9%)	24.4
Weight (kg)	14 (34%)	889 (32.6%)	69.0
% Height/Weight Index	5 (12%)	728 (26.7%)	99.7%
% Ideal Weight	1 (2%)	30 (1.1%)	107.8%
Missing Anthropometrics	2 (5%)	175 (6.5%)	N/A

^a^Number of studies that reported variable of interest out of total of 41 studies.

^b^Weighted by the sample size reported for variable of interest.

*N/A* Not applicable.

## Results

### Search strategy

**Fig. 1:**
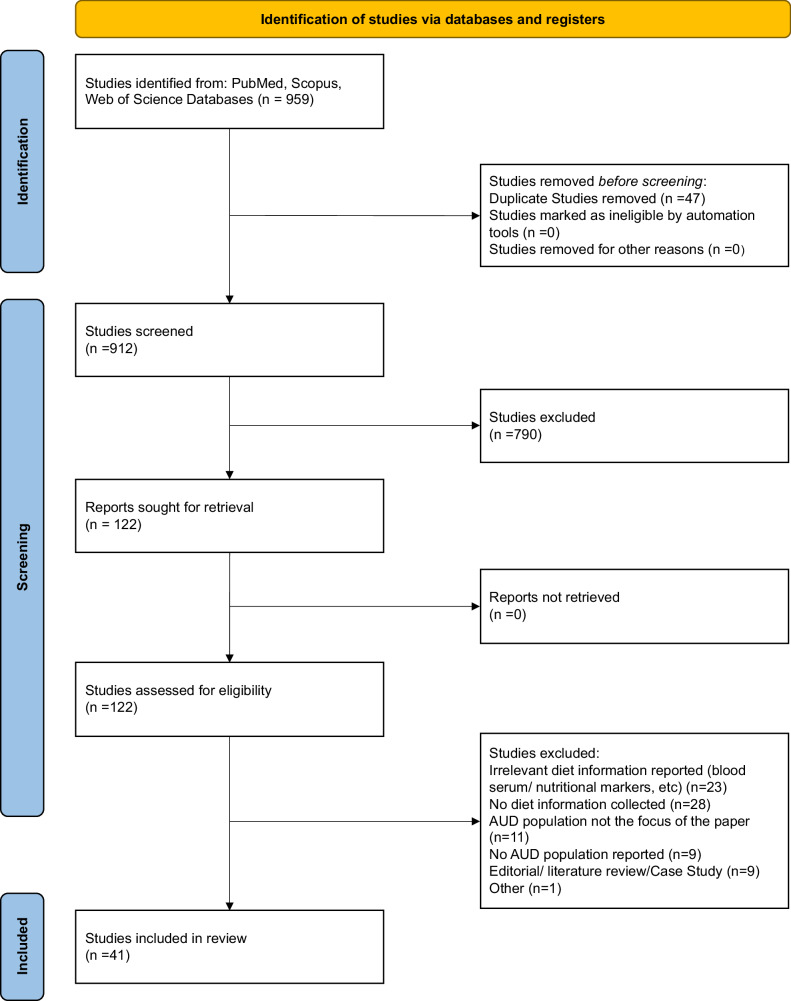
PRISMA flow chart for study selection. Study flow chart using PRISMA guidelines for study selection and screening.

Our search identified 959 potential records. After removing duplicates, 912 records remained for screening. Four reviewers screened the titles and abstracts of the 912 studies using Covidence (*Covidence*, 2023). Following the initial screening, 790 papers were excluded, leaving 122 papers for full-text review and 41 studies were included for data extraction (Fig. 1). The updated search performed in July 2024 did not add any new studies. Extracted data are included in Supplemental Table S1.

### Study characteristics

**Fig. 2:**
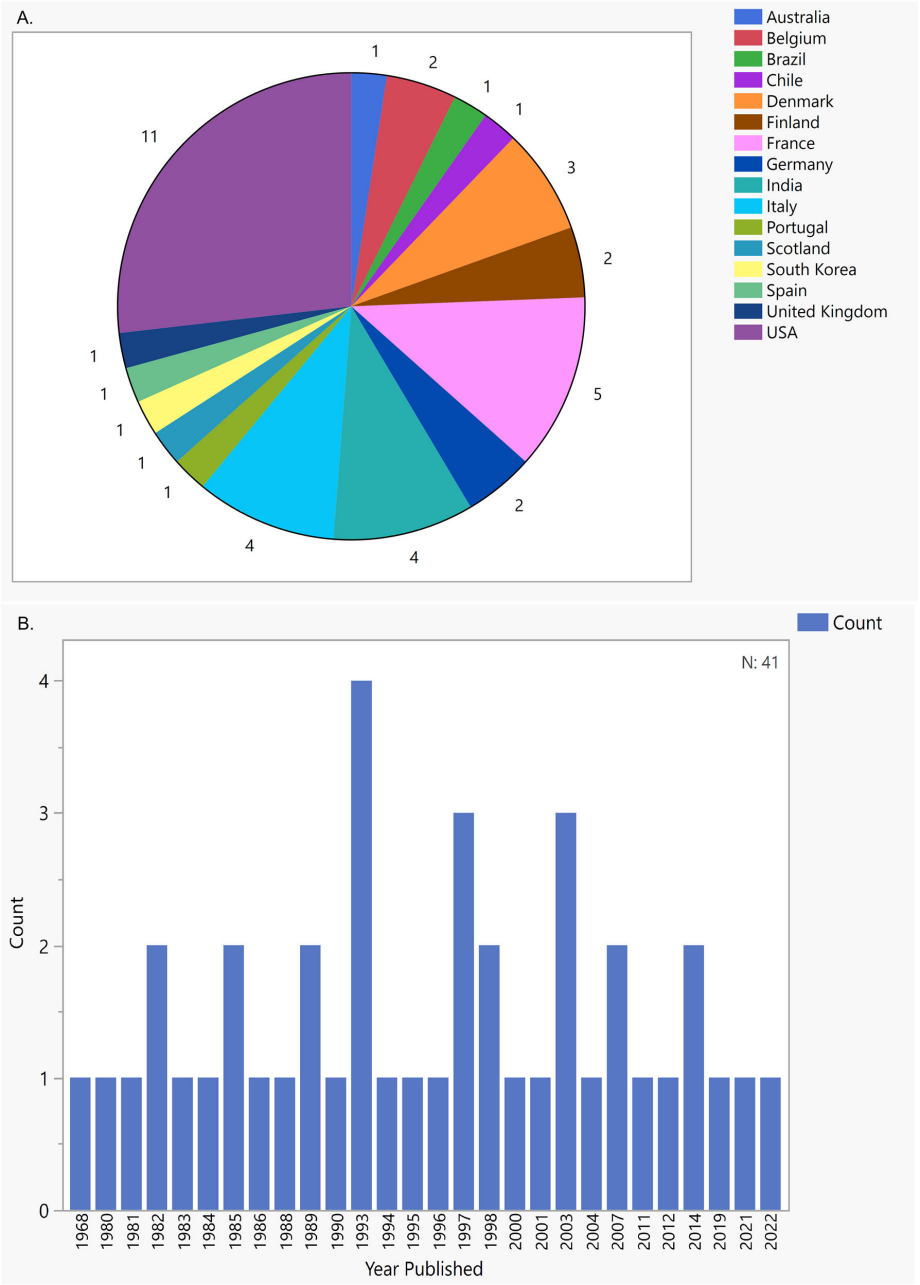
Characteristic distributions of the included studies. Regional and chronological distribution of the included studies. **A** The regional distribution of studies published across 16 countries. **B** The distribution of the number of published studies (Y-axis) by year between 1968 to 2022 (X-axis).

This scoping review includes 41 studies conducted across 16 countries with the United States being the most represented (11 studies), followed by France (5 studies), Italy (4 studies) and India (4 studies) (Fig. 2A). The included studies were published between 1968 and 2022 (Fig. 2B). The earliest, in 1968, evaluated the nutritional status of 34 individuals with AUD through dietary interviews and assessment of vitamin levels via urinalysis [22]. To our knowledge, this study was the first investigation of its kind, with the next study on this topic not published until 1980 [23].

### Sample population demographics and alcohol-related diagnoses

A total of 2727 individuals with AUD and/or alcohol misuse were included in this scoping review. For the studies that reported participant sex, the majority were male (N = 2064, 75.7%) (Table 1). The average age range was between 38–54 years (Table 1). Of the studies that reported BMI (19 studies; 46%), nearly all participants were within the normal BMI range (18.5–24.9 kg/m^2^), with a weighted mean of 24.4 kg/m^2^. The remaining studies reported only weight as kilograms or pounds (14 studies), a height to weight ratio (5 studies), or percent of ideal body weight (1 study) (Table 1).

Participants represented a broad spectrum of AUD and related conditions. Diagnoses were primarily based on evaluation of clinical evaluations including assessment of symptoms using the Diagnostic and Statistical Manual of Mental Disorders, observation of clinical signs (e.g., presence of liver disease or pancreatitis) or responses to diagnostic questionnaire (Supplemental Table S4). A number of studies included participants with alcohol-associated liver disease [24–29], encompassing a broader range of alcohol-induced health complications beyond liver damage alone.

### Assessing dietary intake in people with AUD or AUD-related diagnoses

Dietary intake patterns in individuals with alcohol misuse are presented with a specific focus on two phases: 1) during active alcohol use (assessed retrospectively) and 2) during early abstinence from alcohol. For reference, diet indices are reported alongside those of the typical 20 and over aged American based on What We Eat in America (WWEIA) tables [30]. All weighted averages are shown in Table 2.

**Table 2:** Dietary energy and macronutrient intake during active drinking and abstinence.

Extracted Data	Total Sample Size (N); # of studies^a^	Total Sample Weighted Average^b^	Active Drinking^c^ Sample Size N^a^	Active Drinking Weighted Average^b^	Abstinent^d^ Sample Size N^ab^	Abstinent^d^ Weighted Average^bb^	Reference Sample WWEIA^e^ Average
Body Mass Index (BMI) (kg/m^2^)	1115 (19/41)	24.4	1056 (17/34)	24.4	59 (2/5)	23.0	NR
Total Energy (kcal)	2489 (35/41)	2849	2294 (30/34)	2894	166 (4/5)	2320	2036
Total Energy (excluding alcohol) (kcal)	1843 (29/41)	1956	1685 (25/34)	1928	129 (3/5)	2384	NR
Total Energy from alcohol (kcal)	289 (7/41)	1430	260 (6/34)	1539	0	0	56.7
% kcal from Carbohydrates	875 (12/41)	37.7	783 (10/34)	36.8	92 (2/5)	45.2	45.0
% kcal from Protein	875 (12/41)	13.6	783 (10/34)	13.1	92 (2/5)	18.2	16.0
% kcal from Fat	875 (12/41)	29.4	783 (10/34)	28.5	92 (2/5)	36.5	37.0
Total Alcohol (g)	1981 (24/34)	190.2	1981 (24/34)	190.2	0	0	8.1
Total Carbohydrate (g)	847 (15/41)	266.4	818 (14/34)	265.8	0	NR	228.0
Total Protein (g)	1179 (22/41)	74.3	1113 (20/34)	75.3	37 (1/5)	54.0	77.1
Total Fat (g)	894 (16/41)	79.5	865 (15/34)	79.7	0	NR	85.8
Total Fiber (g)	379 (5/41)	18.0	357 (4/34)	17.6	22 (1/5)	25.8	16.3

^a^Number of studies that reported variable of interest out of total of 41 studies.

^b^Weighted by the sample size reported for variable of interest.

^c^ Refers to diet assessed during active drinking (real-world) setting.

^d^Refers to diet assessed during abstinence period.

^e^**WWEIA**: data derived from WWEIA/NHANES 2021–2023 data Table 1 from the “20 and over” row of the “Males and Females” section.

*g* grams, *kcal* kilocalorie, *NR* Not reported.

### Dietary intake during active drinking

Thirty-six studies assessed diet during active drinking (Supplemental Table S1 and Table 2**)**. Across the 2294 participants of the studies reporting dietary intake during active drinking, the average weighted total daily energy intake was 2894 kcal. Alcohol contributed to a daily average of 1539 kcal, or ~15 standard drinks/day. Participants reported consuming a lower proportion energy from carbohydrates than the general U.S. population (average of 36.82 vs. 45% of calories) though absolute carbohydrate intake was greater (266 g/day vs. 228 g/day). Reported protein intake accounted for 13.1% of energy, and fat for 28.5% of total energy consumed, reflecting a lower proportion of energy from fat when compared to the U.S. population average of 37%. Fiber intake was reported in only four of the 36 studies, with an average daily intake of 17.6 g, which, while similar to typical U.S. consumption (16.3 g/day), is below recommended intakes (U.S. Department of Health and Human Services & U.S. Department of Agriculture, 2020).

### Dietary intake during abstinence

Five studies assessed diet during abstinence (Supplemental Table S1 and Table 2**)**. Overall, these studies reported adequate caloric and macronutrient intake, when compared to a healthy reference population or the Recommended Dietary Allowances (RDA). However, participants’ diets were consistently deficient in specific micronutrients [9, 31, 32]. Of the five studies, two focused on people with AUD and malnutrition risk, and these found that participants under-consumed macronutrients, micronutrients, and total energy [33, 34]. One study assessed malnutrition risk using the Detsky index [34] and the other used serum leptin levels in people with liver disease [33]. Four of the five studies were during an inpatient treatment setting [9, 31, 33] and the other study was conducted in a residential long-term treatment program [32]. Average weighted daily reported energy intake was 2320 kcal/day with an average 18.2% of energy from protein, 45.2% of energy from carbohydrates and 36.5% of energy from fat (only one study reported absolute protein intake per day at 54 g/day). Fiber intake was reported in only one study and was higher than the American average of 16.3 g/d at 25.8 g/day, though it should be noted that this study was conducted in an inpatient hospital setting [9].

### Assessing diet quality and alignment with recommended nutrient intake

Of the 41 included studies, only four (9.8%) presented some type of diet quality or nutrient intake assessment [9, 32, 35, 36]. This indicates a significant gap in the literature, suggesting that evaluation of the quality of diets is rarely prioritized. In a sample from an outpatient rehabilitation program, self-reported intake of B vitamins, magnesium, iron, and zinc were below RDA recommended intake, however, all other nutrients met recommended thresholds. Another study assessed diet as the primary outcome during active drinking and employed the United Kingdom’s Recommended Nutrient Intakes (RNI) metric as a diet quality assessment [36]. The RNI outlines daily recommendations for various nutrients for the general adult population and this study included participants with chronic alcohol-associated pancreatitis, a condition that often alters dietary intake and nutrient absorption, not necessarily reflecting the AUD population without pancreatitis [37]. While most of the 30 participants with chronic alcohol-associated pancreatitis met their RNI for daily energy requirements, 51% of their total energy intake came from alcohol. The third study examined reported diet quality during active drinking and employed a contemporary method for evaluating diet quality using the Nova classification system by addressing the consumption of ultra-processed foods (UPFs) [38]. This study found that the actively drinking cohort exhibited a significantly higher Nova score and greater UPF consumption compared to healthy controls [35].

Finally, a fourth study assessed diet quality during early abstinence using the Healthy Eating Index-2015 in a sample of individuals with AUD undergoing inpatient treatment [9, 39]. Diets consumed during this period were comparable in macro- and micronutrient composition to those of the general U.S., population but demonstrated higher diet quality (HEI-2015: 62 ± 11 vs 58 ± 14, respectively) [40]. The participants of the study self-selected meals from a hospital menu without dietary constraints.

### Dietary assessment tools used for populations with AUD

**Fig. 3:**
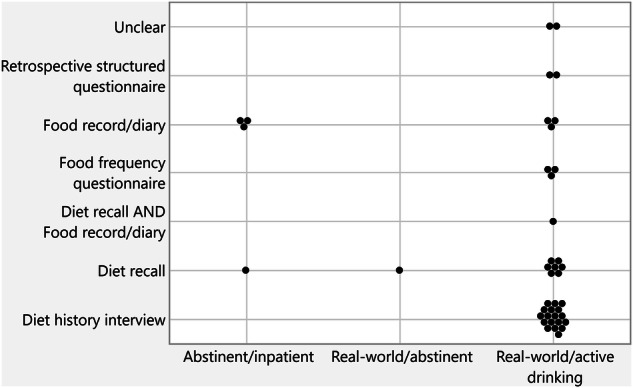
Type of dietary assessment used across patient drinking status. A dot plot showing the diet assessment tools employed during different settings either as abstinent or during active drinking. The primary assessment tool employed was a diet history interview represented across 18 studies.

Because of the potential for recall bias, we included an overview of the dietary assessment tools used in the 41 studies evaluating dietary intake behaviors of individuals with AUD during different stages, such as abstinence/inpatient care, abstinence/real-world, and active drinking/real-world (Fig. 3, Table 3, Supplemental Table S1 and S5**)**. Diet assessments included a range of dietary assessment timeframes including over the past year, month, 7 days or 24 h. Among the five studies assessing diet during abstinence, two used a diet recall [31, 32] and three used food records and/or food diary assessments [9, 33, 41].

**Table 3:** Number and proportion of studies which employed the different diet assessment tools.

Diet Assessment Tool Name	Number of Studies (N = 41)
Diet History Interview	18 (43.9%)
Diet Recall	9 (22.0%)
Food Record/Diary	6 (14.6%)
Food Frequency Questionnaire	3 (7.3%)
Retrospective Structured Questionnaire	2 (4.9%)
Unclear	2 (4.9%)
Diet Recall and Food Record/Diary	1 (2.4%)

Total number of studies that used different dietary assessment tools.

Among the active drinking group, the most used assessment tool was the diet history interview, which was employed in 18 studies (43.9%). Other frequently used methods among the active drinking group included diet recall (7 studies) [25, 35, 36, 42–45], food record/diary (3 studies) [46–48], and a food frequency questionnaire (FFQ) (3 studies) [18, 49, 50]. A retrospective structured questionnaire was used in two other studies [51, 52]. Notably, there were two studies that reported “unclear” methods for dietary assessment during real-world/active drinking [26, 34].

## Discussion

This scoping review and meta-analysis is, to our knowledge, the first comprehensive evaluation of diet-related intake, diet quality, and assessment tools among individuals with AUD and/or alcohol-related diagnoses. By combining dietary intake data across studies, we report weighted averages comparing abstinent individuals and those actively consuming alcohol. As diet quality reflects overall dietary health, including variety, nutrient density, and guideline adherence, these findings offer a foundation for integrating nutrition more effectively into AUD treatment and recovery.

### Dietary intake in active vs. abstinent individuals with AUD

As expected, the most apparent difference between actively drinking and abstinent individuals assessed is alcohol consumption. The active drinking sample consumed a very large amount of alcohol (1539 kcal/day on average), representing a significant proportion of their total caloric intake. In addition to contributing to greater overall energy intake and increasing risk of weight gain, this consumption pattern may result in nutrient imbalances and various health risks [53]. Interestingly, even though this group consumes a significant number of calories from alcohol, energy intake from foods and non-alcohol beverages does not seem to be vastly displaced based on the literature reviewed here. The average energy intake excluding alcohol in the active drinking group was 1928 kcals, and in the abstinent group was 2384 kcals which suggests that alcohol calories are consumed in addition to food and non-alcohol beverage intake during active drinking. Though the active drinking group derives a comparatively smaller percentage of calories from protein (13.1 vs 18.2%), absolute protein intake appeared to be higher in the active drinking group compared to abstinent (75 g/day and 54 g/day respectively), though only one study reported total grams of protein in the abstinent group. Considering similar weighted average body weight in both groups (Table 1), this level of protein intake would be sufficient to achieve the RDA of 0.8 g of protein/kg body weight that is recommended for the general healthy population. Additionally, the abstinent sample reported a higher fiber intake (25.8 g/day) compared to the active drinking sample (17.6 g/day). However, fiber intake was reported in only one study in the abstinent sample so this comparison should be interpreted with caution [9]. This result may reflect the structured and potentially healthier dietary environment of the inpatient setting. The active drinking group reported lower dietary fat as a percentage of total calories (28.5%) compared to the abstinent group (36.5%), but both groups are within or near the acceptable range of fat intake (20–35% of energy). However, it is unclear from these data whether reported diets differ in dietary fat composition (saturated, monounsaturated, polyunsaturated fat), which have important implications for health outcomes such as liver, cardiovascular and metabolic diseases [54].

Overall, individuals in the active drinking group report altered distribution of energy from macronutrients due to the addition of alcohol. As the percent of energy from alcohol increases, the percent of energy from carbohydrate, protein, and fat decreases, which makes comparison to an abstinent group difficult. Of the 36 studies examining diet during active drinking, 22 of them were specifically designed to assess reports of overall dietary intake habits during active drinking phases. These 22 studies broadly found that individuals with AUD consumed adequate calories, but, naturally, a substantial proportion of their caloric intake was derived from alcohol. Additionally, compared to the average US adult, dietary intake patterns of abstinent individuals living inpatient or in residential treatment settings show some notable differences including a higher total caloric intake than the average American adult (2320 kcal/day vs. 2036 kcal/day, respectively). We speculate that the slightly greater energy intake may have resulted from these individuals replacing alcohol cravings with food but additional research on this area is needed to confirm. The macronutrient distribution is relatively similar between the two groups, though the abstinent sample has a slightly higher proportion of calories from protein than the average American adult. It is worth noting that factors affecting the dietary intake of the average American, such as food budget and food preparation capabilities, are less relevant in inpatient treatment settings and may impact this comparison of intake.

Of note, the average weighted BMI of the included sample was 24.4 kg/m^2^, which indicates a BMI in upper range of normal. Given that the average dietary intake reported across the studies assessing diet in early abstinence appeared to reflect generally adequate or “normal” consumption patterns, this suggests a low likelihood of primary malnutrition due to under nutrition, caloric insufficiency or excess renal excretion. Despite this, nutritional deficiencies in people with AUD are consistently reported and clinically relevant [4, 7]. It is therefore possible that these deficits are more likely to be a potential result of secondary malnutrition, specifically, alcohol-induced impairments in nutrient digestion, absorption, metabolism, and utilization and excess excretion [4, 55]. Mechanisms by which alcohol affects nutrient utilization include alcohol-induced gastritis and intestinal inflammation that impair digestion and absorption of essential nutrients [56, 57], disruption of hepatic metabolism leading to reduced enzymatic activation of vitamins such as thiamine and folate [58], and increased urinary excretion of micronutrients including magnesium, zinc, and B vitamins [59]. This distinction is critical because it implies that standard dietary guidelines developed for the general U.S. population may not adequately address the unique nutritional needs of individuals with AUD. Therefore, there is a clear need for further research to define AUD-specific nutritional requirements and, ultimately, to develop revised dietary reference intakes or clinical recommendations tailored to this high-risk population. Interestingly, despite potentially high total caloric intake, including calories from alcohol, participants with active AUD generally fell within the normal BMI range. This may reflect several factors. Although alcohol provides substantial calories, these often replace nutrient-dense foods rather than add to total intake. Additionally, alcohol calories are metabolized less efficiently, and chronic alcohol use can increase energy expenditure and impair nutrient absorption and utilization [60]. Together, these mechanisms may contribute to a relatively normal BMI despite high reported energy intake. It is also possible that these effects on energy metabolism normalize within 3–6 months after abstinence [61] highlighting the need for further investigation of how nutrient requirements may vary over the course of recovery. Additionally, underreporting of dietary intake remains a potential limitation. These findings suggest that body weight alone may not be a reliable indicator of nutritional status in individuals with AUD and highlight the need for careful assessment of both dietary intake and nutrient status. Furthermore, it is also important to keep in mind that BMI does not differentiate between fat mass and fat-free mass, nor does it account for differences in fat distribution, therefore BMI can underestimate and overestimate adiposity [62].

### The reporting of daily fiber intake is scarce in the literature

As a key component of a healthy diet, dietary fiber plays a critical role in maintaining gastrointestinal health, regulating blood glucose, supporting cardiovascular function, and modulating the gut microbiome balance, amounting to benefits on overall physical and mental health [63–65]. Chronic alcohol consumption is associated with nutrient deficiencies, including depleted stores of B-vitamins, zinc, and magnesium [10, 11], all of which are often found in fiber-rich foods. Thus, increasing intake of fiber-rich foods during recovery may provide dual benefits: supporting intestinal health through improved gut function and microbiome composition, while simultaneously replenishing micronutrients critical for neural and metabolic processes [66]. These nutrients contribute to mitochondrial ATP production, neurotransmitter synthesis, and the prevention of excitotoxicity, thereby linking intestinal and mental health outcomes [66]. Five studies included fiber as part of their dietary intake assessments in individuals with AUD, four of which were during active drinking status. Of the four studies reporting dietary fiber intake during active drinking, the evidence is mixed with two studies reporting below the US average daily fiber intake of 16 g/day [35, 43] and two studies reporting above the average intake of fiber [41, 50]. In early abstinence, reported total daily fiber intake appears to be above average at 25.8 g/day [9, 30], though this may be due to availability and access to a balanced diet during inpatient treatment as well as a diet structured to support digestive health and overall nutritional needs. Given that only 5 out of the 41 studies reported total fiber intake, this represents a gap in the literature and this metric should be considered in future studies assessing diet in people with AUD.

### Diet quality and nutrient assessment among people with AUD: significant gap in the literature

Diet quality measurement is used to evaluate the overall healthfulness of an individual’s diet and alignment with dietary guidelines. It provides a summary measure of overall diet patterns, rather than focusing only on individual nutrients or foods, and helps determine whether a person is eating in a way that supports health and reduces disease risk. Despite the documented nutritional impairments observed in AUD, this scoping review highlights a striking lack of studies that rigorously assess diet quality in this population with the limited data available. Only four of the 41 included studies (9.8%) evaluated the diets either by quality or by alignment with references of nutrient intake among individuals with AUD in comparison to an established reference population or to composite indices of dietary patterns associated with health. This limited focus may reflect historical research priorities that centered more on caloric intake or nutrient deficiencies rather than a holistic view of dietary patterns. Accordingly, earlier studies used national dietary reference standards (RDA, RNI), highlighting nutrient-specific insufficient intakes but not capturing the broader structure or quality of the diet [32, 36]. More recent studies have incorporated standardized measures of diet quality, such as the Healthy Eating Index (HEI) or Nova classification, to evaluate the extent to which dietary patterns align with established nutritional guidelines and predict long-term health outcomes [38, 39, 67]. Notably one study utilizing the HEI found that individuals undergoing inpatient treatment for AUD consumed diets of higher quality than the general U.S. population, potentially reflecting the influence of structured and supportive food environments in inpatient clinical settings [9]. Conversely, in another study conducted in Belgium that used the Nova system indicated that individuals with AUD in an active drinking state may consume a disproportionately high share of UPF, which are linked to poor metabolic and mental health outcomes [68]. Of note, the latter study may be context-specific, e.g., specific to the Belgian population; for example, a more recent study in the U.S., conducted by our team and published after this scoping review was completed, did not find differences in diet quality nor in UPF consumption between people with AUD vs. controls, likely due to a ceiling effect reflecting the high consumption of UPFs among the American population in general [69]. Collectively, these studies suggest diet quality may depend on treatment status. The limited number of studies using formal diet quality metrics suggests a clear gap in the literature and emphasizes the need for further research in this area.

### Dietary assessments in populations with AUD and the need for a validated tool

The findings of this scoping review reveal significant heterogeneity in dietary assessment tools used to evaluate nutritional intake among individuals with AUD, with methods varying based on drinking status and living conditions. While structured tools like the diet history interview was most used (n = 18), particularly among individuals with active alcohol use in real-world living conditions, other studies employed diet recalls, food records, food frequency questionnaires, and two studies provided undefined dietary intake assessment methods. This variation in approach reflects a lack of consensus in the field and poses a challenge for drawing comparisons across studies.

The diet history interview allows for detailed, interviewer-guided recall of long-term dietary habits, but its reliance on memory poses challenges, particularly in individuals with AUD [18]. While dietary assessment tools continue to be refined for general populations [8, 70], none have been specifically validated for use in individuals with AUD. For instance, one study noted that participants with AUD had difficulty accurately recalling intake using the Diet History Questionnaire II, a standardized FFQ [18, 71]. These findings highlight the need for standardized, validated tools tailored to the cognitive and behavioral profiles of people with AUD, which would improve data accuracy, enable cross-study comparisons, and strengthen the evidence linking diet to alcohol-related health outcomes.

### Limitations

A major limitation of this scoping review was the variability in how dietary intake data were reported across the 41 included studies. Nutrient and energy intake were presented in differing units (e.g., kilocalories, grams, percent energy), requiring extensive harmonization. Despite standardization efforts **(**Supplementary Table S3), some datasets were excluded due to missing or incompatible variables, such as absent participant weight or unclear units [49, 72]. Additionally, individual energy needs were not accounted for, potentially affecting interpretations of adequacy across diverse populations. Missing data further limited comprehensiveness, with omissions ranging from a few studies to over 30 per variable (Supplementary Table S1). Inconsistent reporting of anthropometrics (e.g., BMI vs. ideal weight indices) and macronutrients (e.g., grams/day vs. % total energy) complicated comparisons. While harmonization reduced some inconsistencies, excluded or incomplete datasets may have impacted the precision of summary estimates. These findings underscore the need for standardized reporting in future nutrition research on alcohol misuse.

### Conclusions and recommendations

Nutrition profoundly influences both physical and mental health, making it an essential but underleveraged component of AUD treatment and recovery[4]. Poor diet quality can exacerbate inflammation, cognitive dysfunction, and comorbid conditions often present in people with AUD, while improved nutritional intake, particularly diets rich in fiber, micronutrients, and balanced macronutrients, may support physiological healing, psychological well-being and recovery [4, 7]. To our knowledge, only a few clinical trials have been reported on dietary interventions in AUD [16, 73–75]. However, this review provides a foundation on which to focus future intervention trials on determining what level of nutrient intake and type/quantity of specific dietary patterns may optimally correct nutrient deficiencies in this population. This work highlights critical gaps and inconsistencies in how dietary intake is assessed, reported, and interpreted in individuals with AUD. The diversity in dietary assessment tools, lack of standardized reporting units, and frequent missing data across studies severely limit the ability to compare findings or draw evidence-based conclusions. These methodological disparities highlight an urgent need for unified approaches to dietary data collection for this population. Without standardized protocols, it remains challenging to develop actionable nutritional guidelines or to understand how dietary patterns influence the progression, recovery, or comorbidities associated with AUD. This review also serves as a call to action advocating for (1) the consistent and systematic investigation of dietary habits in individuals with AUD, (2) the harmonization of dietary assessment tools that allow for cross-study comparability and global relevance and (3) a need to define AUD-specific nutritional requirements and to develop revised dietary reference intakes or clinical guidelines tailored to this high-risk group. Such guidelines may warrant particular attention to micronutrients that are commonly deficient in this population, including B vitamins (e.g., thiamine, folate, vitamin B6, and B12), and minerals such as magnesium and zinc, given their roles in alcohol metabolism, neurological function, and overall recovery. Furthermore, as the field continues to move toward understanding the relationship of the gut-brain axis and the impact that the gut microbiome has on addiction neuroscience including AUD, standardized and comprehensive assessment of nutritional intake will be essential [76–78]. Integrating nutritional science into the broader framework of AUD research is vital, not only to improve treatment outcomes but also to unlock new therapeutic pathways at the intersection of diet, microbiota, and brain function.

Future research should prioritize the development of validated, AUD-specific dietary assessment instruments that reflect the cognitive and behavioral complexities of this population. Such efforts could inform nutritional interventions aimed at improving recovery outcomes and reducing comorbidities in this vulnerable group and will be crucial to integrate nutrition into the broader framework of AUD prevention, treatment, and recovery strategies, bridging a major gap in both clinical research and care.

## Supplementary information

Supplemental Material

Supplementary Tables

## Data Availability

The data and literature search protocol underpinning this paper can be requested from the corresponding authors.
