# Supplementary material for: Dietary intake, quality, and assessment tools in individuals with problematic alcohol use: a scoping review and meta-analysis
Source: Transl Psychiatry. 2026 Jan 28;16:51. doi: 10.1038/s41398-026-03842-9 (PMC12873200; doi:10.1038/s41398-026-03842-9)
Supplement: Supplementary file 1 — Supplemental Material [file 41398_2026_3842_MOESM1_ESM.docx]

Supplemental Material and Methods:

**Supplemental Methodology:**

Three databases were searched: PubMed/MEDLINE, Scopus and Web of Science using a combination of Medical Subject Headings (MeSH) terms and keywords procedure. Two searches were conducted: one in October 2023 with no date limitations and an update in July 2024. Four authors performed title and abstract screening to generate the list of full text review, and nine authors performed full text review and data extraction for all included studies. Covidence was used for title and abstract screening and full text review (Covidence systematic review software, Veritas Health Innovation, Melbourne, Australia. Available at www.covidence.org). The extracted data were compiled, cleaned, reviewed and transcribed into one table for reporting. This scoping review followed the Preferred Reporting Items for Systematic reviews and Meta-Analyses extension for Scoping Reviews guidelines for conducting a scoping review (PRISMA-ScR). The scoping review protocol was preregistered with the Open Science Framework (<https://osf.io/y6mx7>) (1,2).

**Scoping Review Framework**

| 1. Research question identification | Group meetings to identify and clarify the research questions and aims for the scoping review |
| --- | --- |
| 2. Identify all studies that are relevant to the study question | Three databases (Medline, Scopus, and Web of Science) were queried with constructed MESH terms and studies were selected based on inclusion criteria. |
| 3. Relevant study selection | Selection of studies using a PRISMA approach. Four team members screened abstracts and manuscripts for study relevance and conflicts were resolved. All abstract screening was conducted by at least by two team members. |
| 4. Data extraction and data presentation | The data from relevant studies was charted in a spreadsheet in Microsoft Excel with all relevant information to address the review aims |
| 5. Collating and dissemination of results | Data was extracted from each study and converted to a concordant metric across all studies. |

*Identifying the relevant questions*Individuals with AUD tend to consume most of their energy in the form of alcohol and may be at high risk of consuming nutritionally deficient diets (3,4). Most studies assess the nutritional status of individuals with AUD using anthropometric measurements and blood serum markers and find that individuals with AUD report a malnourished state (4). However, dietary intake behaviors in people with AUD are rarely assessed. The primary aim of this scoping review is to collate and report all studies who report the diet intake behaviors across the patient population. A secondary goal of this review was to survey the differing dietary assessment tools used in clinical research for individuals with AUD especially during periods of heavy drinking when diet recall may be impaired. Given the limited existing research, a scoping review with a quantitative approach was chosen over a systematic review. This method is appropriate for mapping the current state of research in a field, particularly when the area encompasses diverse study methodologies or when it represents the first comprehensive consolidation of research. The key questions addressed are as follows:

1. *What are the typical dietary intake patterns of individuals with AUD during active drinking and abstinence (e.g., macro and micronutrient intake and dietary quality)?*
2. *What types of diet quality metrics are reported for individuals with AUD?*
3. *What tools are used to assess dietary intake in individuals diagnosed with AUD?*

*Identifying the relevant studies*To execute the searches of the three databases, a list of terms was developed through discussions with the team which included a Library Informationist. Database searches were conducted on October 3, 2023 and updated on July 25, 2024. All search terms used are shown below with their searched database.

*Search terms and databases used for primary searches*
Database: PubMed/MEDLINE
Platform: National Library of Medicine
Date Searched: 10/3/2023
Updated Search: 10/3/2023 - 7/25/2024

|  | Concept | Search Strategy |
| --- | --- | --- |
| #1 | Nutrition | "Nutritional status"[Mesh] OR "Feeding Behavior"[Mesh] OR "Eating"[Mesh] OR "eating habit*"OR "Diet Records"[Mesh] OR "Diet"[Mesh] OR "diet* intake*" OR "Nutrition Assessment"[Mesh] OR "nutritional status"[Title/Abstract] OR "dietary status"[Title/Abstract] OR "dietary habit*"[Title/Abstract] OR "feeding behavior*"[Title/Abstract] OR "food intake*"[Title/Abstract] OR "dietary intake*"[Title/Abstract] OR "nutritional intake*"[Title/Abstract] OR "diet* record*"[Title/Abstract] OR "food diary"[Title/Abstract] OR "food diaries"[Title/Abstract] OR "food consumption"[Title/Abstract] OR "ultra processed food*"[Title/Abstract] OR "ultra processed food*"[Title/Abstract] OR "ultraprocessed food*"[Title/Abstract] |
| #2 | Alcoholism | "Alcoholism"[Mesh] OR "Alcohol-related disorders"[Mesh] OR "alcohol dependenc*"[Title/Abstract] OR "alcohol addict*"[Title/Abstract] OR "alcohol abuse*"[Title/Abstract] OR "alcohol use disorder*"[Title/Abstract] |
| #3 | Limits | #1 AND #2 Filters: English, from 2023/10/3 - 2024/7/23 |

­­

Database: Web of Science (Core Collection)
Platform: Clarivate Analytics

Date Search 10/2023
Updated Search: 10/3/2023 - 7/25/2024

|  | Concept | Search Strategy |
| --- | --- | --- |
| #1 | Nutrition | TS=("nutritional status" OR "feeding behavior*" OR "eating habit*" OR (diet* AND record*) OR diet* habit* OR "nutrition assessment*" OR "nutritional status" OR "dietary status" OR "dietary habit*" OR "food intake*" OR "dietary intake*" OR "nutritional intake*" OR "food diary" OR "food diaries" OR "food consumption*" OR "ultra processed food*" OR "ultra-processed food*" OR "ultraprocessed food*") |
| #2 | Alcoholism | TS=(alcoholism OR "alcohol-related disorder*" OR "alcohol dependenc*" OR "alcohol addiction" OR "alcohol abuse" OR “alcohol use disorder*”) |
| #3 | Limits | #2 AND #1 and English (Languages) \| Timespan: 2023-10-03 to 2024-07-23 (Publication Date) |

­­

Database: Scopus
Platform: Elsevier
Date Searched: 10/3/2023

Updated Search: 10/2023 - 7/2024

|  | Concept | Search Strategy |
| --- | --- | --- |
| #1 | Nutrition | ( TITLE-ABS-KEY ( "nutritional status" OR "feeding behavior*" OR "eating habit*" OR ( diet* AND record* ) OR (diet* AND habit*) OR "nutrition assessment*" OR "nutritional status" OR "dietary status" OR "dietary habit*" OR "food intake*" OR "dietary intake*" OR "nutritional intake*" OR "food diary" OR "food diaries" OR "food consumption*" OR "ultra processed food*" OR "ultra-processed food*" OR "ultraprocessed food*" ) |
| #2 | Alcohol Use Disorder | TITLE-ABS-KEY ( alcoholism OR "alcohol-related disorder*" OR "alcohol dependenc*" OR "alcohol addiction" OR "alcohol abuse" OR "alcohol use disorder*" ) ) |
| #3 | Limits | ( TITLE-ABS-KEY ( "nutritional status" OR "feeding behavior*" OR "eating habit*" OR ( diet* AND record* ) OR diet* AND habit* OR "nutrition assessment*" OR "nutritional status" OR "dietary status" OR "dietary habit*" OR "food intake*" OR "dietary intake*" OR "nutritional intake*" OR "food diary" OR "food diaries" OR "food consumption*" OR "ultra processed food*" OR "ultra-processed food*" OR "ultraprocessed food*" ) AND TITLE-ABS-KEY ( alcoholism OR "alcohol-related disorder*" OR "alcohol dependenc*" OR "alcohol addiction" OR "alcohol abuse" OR "alcohol use disorder*" ) ) AND ( LIMIT-TO ( LANGUAGE , "English" ) ) AND ( LIMIT-TO  ( DOCTYPE , "ar" ) OR LIMIT-TO ( DOCTYPE , "re" ) ) AND ( LIMIT-TO  ( SRCTYPE , "j" ) ) |

­

***Inclusion and exclusion criteria***

Criteria for relevant studies for inclusion were listed as follows: English language documents; studies involving human participants; individuals with an AUD diagnosis, alcohol-related disease, and/or high drinking levels suggestive of AUD; and at least one measure of dietary intake assessment or diet quality in the absence of a dietary intervention. The included sample will be described as individuals with AUD throughout this work. Exclusion criteria included: non-English documents; non-human participants (*e.g.,* primate or murine models); studies in which participants were not described to have problematic alcohol misuse or AUD; and studies with dietary intake data only in the context of a nutritional intervention with no baseline diet assessment.

*Study selection*
Once the database search was complete and duplicates removed, all abstracts were imported into Covidence screening software for title and abstract review (7). Every imported study was independently reviewed initially by two authors using the study title and abstract to ascertain relevance. For any studies where the relevancy was unclear the first four authors would discuss further based on abstract if a paper could go to full text review. Both authors would have to vote “yes” for the study to progress to full text review. If the abstract was unclear about the data acquired, the study team would refer to the full text to review the study’s methodology and determine if the study adequately reflected the dietary intake of the patient population. If there were any conflicts, where both study team members voted differently, three members of the reviewer committee met to assess and resolve the conflicts. To ensure inter-rater reliability, the team conducted practice reviews on five papers to refine the spreadsheet and standardize interpretations. During the formal review process, 10% of the papers were double reviewed to confirm consistency. Papers were assigned at random, and responses were cross-checked during the creation of the final dataset. In the few cases of disagreement, the papers were re-reviewed to clarify and reach consensus.

*Data extraction and data presentation*Full text review and data extraction happened simultaneously using an external spreadsheet charting process in Microsoft Excel (**see Supplemental Table S1-2**). Prior to starting full text review and data extraction, a custom template was developed through an iterative process to meet the needs of this review. The final data extraction template was evaluated and agreed upon by all reviewers. All reviewers independently extracted data into the final data extraction template and answers were cross-checked to assess inter-rater reliability. This was done in a group meeting to assess concordance across different reviewers; no inter-rater reliability metrics were recorded.

*Collating, summarizing and visualizing the results*Dietary intake was categorized as reflecting intake in a real-world environment during alcohol use or abstinence. After data extraction, specific data conversion methods were applied where necessary to ensure concordance across all included studies (*e.g*., converting kJ/day or MJ/day to kcal/day, or lb. to kg). Any conversions performed are detailed in Supplementary Material (**Supplemental Table S3**). Six studies reported other metrics including, % Height/Weight Index (5 studies) or ‘% Ideal Weight’ (1 study) which is used to compare a person’s actual body weight to the weight that is considered optimal for health based on their height, age, and sex. The % Height/Weight Index metric was a precursor to the commonly used BMI in current times (8–13).

Data visualization and statistical summarization were performed using JMP™ Statistical Discovery software v.16 (SAS Headquarters, Cary, NC). Descriptive statistics, including averages, were calculated using weighted means, where each data point was weighted by its respective sample size. This approach involved multiplying each data point by its sample size, summing these values, and dividing by the total sample size for the variable.

*Alcohol-related diagnoses and questionnaires*
Diagnostic tools to assess alcohol misuse such as questionnaire type, type of alcohol-based disorder, and type of clinical diagnosis were extracted and compiled in **Supplemental Table S4**.

*Comparison to population average intake and dietary recommendation*To facilitate comparison of the dietary intake data to adult (≥20 years of age) American intake averages and national intake recommendations for a healthy diet, we included an assessment of intake averages using the What We Eat in America (WWEIA) tables (**Table 2).** These data report the dietary intake averages of macronutrients, fiber, and alcohol in the typical American compiled from the National Heath and Nutritional Survey (14).  We further provide the recommended intake of macronutrients, fiber and energy provided by the Dietary Guidelines for Americans (DGA) as an additional reference point. The DGA are recommendations provided by the United States Departments of Agriculture and Health and Human Services to promote healthy dietary habits (15).

*Dietary assessment tools*The type of dietary assessment conducted in the included studies were cataloged to better understand the methodologies for dietary evaluation in this population. Tools were categorized into: retrospective structured questionnaires, food records/diaries, food frequency questionnaires, diet recalls and diet history interviews. For any manuscript where the type of assessment type was not explicitly described, the categorization was listed as unclear.

**References**

1. PRISMA-P Group, Moher D, Shamseer L, Clarke M, Ghersi D, Liberati A, et al. Preferred reporting items for systematic review and meta-analysis protocols (PRISMA-P) 2015 statement. Syst Rev. 2015 Dec;4(1):1.

2. Tricco AC, Lillie E, Zarin W, O’Brien KK, Colquhoun H, Levac D, et al. PRISMA Extension for Scoping Reviews (PRISMA-ScR): Checklist and Explanation. Ann Intern Med. 2018 Oct 2;169(7):467–73.

3. Barve S, Chen SY, Kirpich I, Watson WH, Mcclain C. Development, Prevention, and Treatment of Alcohol-Induced Organ Injury: The Role of Nutrition. Alcohol Res Curr Rev. 2017;38(2):289–302.

4. Dicken SJ, Batterham RL. The Role of Diet Quality in Mediating the Association between Ultra-Processed Food Intake, Obesity and Health-Related Outcomes: A Review of Prospective Cohort Studies. Nutrients. 2021 Dec 22;14(1):23.

5. Gautron MA, Questel F, Lejoyeux M, Bellivier F, Vorspan F. Nutritional Status During Inpatient Alcohol Detoxification. Alcohol Alcohol. 2018 Jan 1;53(1):64–70.

6. Covidence systematic review software [Internet]. Veritas Health Innovation; 2023. Available from: www.covidence.org

7. Mendenhall C, Bongiovanni G, Goldberg S, Miller B, Moore J, Rouster S, et al. VA Cooperative Study on Alcoholic Hepatitis III: Changes in Protein‐Calorie Malnutrition Associated with 30 Days of Hospitalization with and without Enteral Nutritional Therapy. J Parenter Enter Nutr. 1985 Sep;9(5):590–6.

8. Mendenhall CL, Moritz TE, Roselle GA, Morgan TR, Nemchausky BA, Tamburro CH, et al. A study of oral nutritional support with oxandrolone in malnourished patients with alcoholic hepatitis: Results of a department of veterans affairs cooperative study. Hepatology. 1993 Apr;17(4):564–76.

9. Mezey E, Kolman C, Diehl A, Mitchell M, Herlong H. Alcohol and dietary intake in the development of chronic pancreatitis and liver disease in alcoholism. Am J Clin Nutr. 1988 Jul;48(1):148–51.

10. Mills P, Shenkin A, Anthony R, McLelland A, Main A, MacSween R, et al. Assessment of nutritional status and in vivo immune responses in alcoholic liver disease. Am J Clin Nutr. 1983 Dec;38(6):849–59.

11. Nicolás JM, Estruch R, Antunez E, Sacanella E, Urbano-Márquez A. NUTRITIONAL STATUS IN CHRONICALLY ALCOHOLIC MEN FROM THE MIDDLE SOCIOECONOMIC CLASS AND ITS RELATION TO ETHANOL INTAKE. Alcohol Alcohol [Internet]. 1993 Sep [cited 2024 Mar 4]; Available from: https://academic.oup.com/alcalc/article/28/5/551/132382/NUTRITIONAL-STATUS-IN-CHRONICALLY-ALCOHOLIC-MEN

12. Videla LA, Iturriaga H, Pino ME, Bunout D, Valenzuela A, Ugarte G. Content of Hepatic Reduced Glutathione in Chronic Alcoholic Patients: Influence of the Length of Abstinence and Liver Necrosis. Clin Sci. 1984 Mar 1;66(3):283–90.

13. Centers for Disease Control and Prevention, National Center for Health Statistics. What We Eat In America (WWEIA) Database. 2015 Apr; Available from: https://agdatacommons.nal.usda.gov/articles/dataset/What_We_Eat_In_America_WWEIA_Database/24660126

14. U.S. Department of Health and Human Services, U.S. Department of Agriculture. Dietary Guidelines for Americans, 2020-2025 [Internet]. 9th ed. 2020. Available from: https://www.dietaryguidelines.gov/resources/2020-2025-dietary-guidelines-online-materials
