# Supplementary material for: Dietary intake, quality, and assessment tools in individuals with problematic alcohol use: a scoping review and meta-analysis
Source: Transl Psychiatry. 2026 Jan 28;16:51. doi: 10.1038/s41398-026-03842-9 (PMC12873200; doi:10.1038/s41398-026-03842-9)
Supplement: Supplementary file 2 — Supplementary Tables [file 41398_2026_3842_MOESM2_ESM.docx]

**Supplemental Table S1: Data Extracted for each study included in the Scoping Review**

| **Reference** | **Study sample information** | **Study design and history of alcohol use** | **Demographics** | **Dietary assessment information** | **Energy and alcohol intake** | **Macronutrients**  **Carbohydrate Protein Fat** | | | **Summary and dietary intake findings**  Were participants taking in normal amount of food and macronutrients? |
| --- | --- | --- | --- | --- | --- | --- | --- | --- | --- |
| Addolorato et al., 1997 | **Dietary Period of Assessment during Study:** Real-world  **AUD Population Sample Size:** Total: 12 M: 6 F: 6  **Diet as Primary Outcome**: No | **Study Design:** Cross-Sectional  **Drinking status**: Active   **Diagnosis Type:** Alcohol Dependence (DSM-III)  **Patient Type:** Clinical Diagnosis of AUD | **Race/Ethnicity:** NR  **Age**: 38.7±8.3  **BMI**: 22±3.17 | **Diet tool used**: Food record/diary  **Timeframe of assessment**: 7-day recall  **Diet assessment conductor**: dietician  **Diet Quality Score**: NR | **Energy including alcohol:**  3140±523 kcal/day  **Energy excluding alcohol:**  1810 ±262 kcal/day **Alcohol (g):** 187.5 ± 57.4 | **AUD:**  54.4%±2.95% | **AUD:**  21.2% ±3.01% | **AUD:**  24.4%±2.46% | Participants with AUD showed an increased REE over predicted values and preferential lipid oxidation with respect to controls. |
| Addolorato, et al., 1998 | **Dietary Period of Assessment during Study:** Real-world  **AUD Population Sample Size:** Total:32 M:18 F:14  **Diet as Primary Outcome:** No | **Study Design:** Cross-sectional  **Drinking status**: Active   **Diagnosis Type:** Alcohol Dependence (DSM-III)  **Patient Type:** Clinical Diagnosis of AUD | **Race/Ethnicity:**  N/R  **Age**:40.9±10.7  **BMI**:22.0±2.46 | **Diet tool used**: Food record/diary  **Timeframe of assessment**: 7-day recall  **Diet assessment conductor**: Dietician  **Diet Quality Score**: N/R | **Energy including alcohol:** **mean** 13,460 ± 1670 KJ/day  **Energy excluding alcohol:** mean 7690 ±1460 KJ/day  **Alcohol (g):** 195 ±57.7 | **AUD:**  52.4%±2.87 | **AUD:**  22.7%±1.87 | **AUD:**  24.9%±1.94 | Participants with AUD had lower weight and a higher REE as compared to social drinkers. |
| **Reference** | **Study sample information** | **Study design and history of alcohol use** | **Demographics** | **Dietary assessment information** | **Energy and alcohol intake** | **Macronutrients**  **Carbohydrate Protein Fat** | | | **Summary and dietary intake findings**  Were participants taking in normal amount of food and macronutrients? |
| Addolorato et al., 2000 | **Dietary Period of Assessment during Study:** Real-world  **AUD Population Sample Size:**  Total: 34  M: 34 F: 0  **Diet as Primary Outcome:** No | **Study Design:** Cross Sectional  **Drinking status**: Active  **Diagnosis Type:** Alcohol Dependence (DSM-III)  **Patient Type:** Clinical Diagnosis of AUD | **Race/Ethnicity:**  **N/R**  **Age**: 41.4±9.7  **BMI**:23.1±2.4 | **Diet tool used:** Food record/diary  **Timeframe of assessment**: 3-day recall  **Diet assessment conductor**: Dietician  **Diet Quality Score**: NR | **Energy including alcohol:** NR  **Energy excluding alcohol:** 2320 ±412 kcal/day  **Alcohol (g):** 194±62.4 | **AUD:**  NR | **AUD:**  NR | **AUD:**  NR | While participants seemed to be consuming a roughly normal amount of kcals, participants with AUD had reduced body fat. |
| Amadieu et al., 2021 | **Dietary Period of Assessment during Study:** Real-world  **AUD Population** **Sample Size:** Total: 48 M: 30 F: 18  **Diet as Primary Outcome:** Yes | **Study Design:** Cross-Sectional  **Drinking status**: Active   **Diagnosis Type:** AUD  (DSM-5 and AUDIT)  **Patient Type:** Clinical Diagnosis of AUD | **Race/Ethnicity:**  **N/R**  **Age**: 48.5±11.4  **BMI**: 24±3.3 | **Diet tool used**: Diet recall   **Timeframe of assessment**: 24-hr recall  **Diet assessment conductor**: dietician  **Diet Quality type**: NOVA Score | **Energy including alcohol:** 2500 kcal/day  **Energy excluding alcohol:** 1400 kcal/day  **Alcohol (g):**132±73.1 | **AUD:**  35% | **AUD:**  10% | **AUD:**  30% | When the kcal from alcohol were removed, participants are taking in a significantly reduced/insufficient amount of daily kcal. |
| **Reference** | **Study sample information** | **Study design and history of alcohol use** | **Demographics** | **Dietary assessment information** | **Energy and alcohol intake** | **Macronutrients**  **Carbohydrate Protein Fat** | | | **Summary and dietary intake findings**  Were participants taking in normal amount of food and macronutrients? |
| Bergheim et al., 2003 | **Dietary Period of Assessment during Study:** Real-world  **AUD Population**  **Sample Size:** Total: 76 M: 76 F: 0  **Diet as Primary Outcome:** Yes | **Study Design:** Cross Sectional  **Drinking status**: Active   **Diagnosis Type:**  Alcohol-induced liver disease  **Patient Type:** Physiological Diagnosis of AUD | **Race/Ethnicity:**  **N/R**  **Age**: 46.26±10.34  **BMI**: 24.28±4.50 | **Diet tool used**: Diet History interview  **Timeframe of assessment**: 24-hr recall  **Diet assessment conductor**: Nutritionist  **Diet Quality type**: NR | **Energy including alcohol:** 14240.67 ±4502.95 KJ/day  **Energy excluding alcohol:** 9979.93 ±3184.25 KJ/day  **Alcohol (g):** 143.03±88.66 g/day | **AUD:**  56.17%±9.74 | **AUD:**  13.33%±2.75 | **AUD:**  29.83%±7.99 | When compared to the healthy controls the ALD group did consume more kj/day, but the ALD group was comparable to the HC group in BMI and in consumption of macronutrients. |
| Campillo et al., 2001 | **Dietary Period of Assessment during Study:** Inpatient  **AUD Population Sample Size:** Total: 37 M: 24 F: 13  **Diet as Primary Outcome:** Yes | **Study Design:** Cross Sectional  **Drinking status**: Abstinent   **Diagnosis Type:**  Alcohol- induced Liver cirrhosis  **Patient Type:** Physiological Diagnosis of AUD | **Race/Ethnicity:**  **N/R**  **Age**: 53.3±9.5  **BMI**:22.4±4.1 | **Diet tool used**: Food record/diary  **Timeframe of assessment**: 24-hr recall  **Diet assessment conductor**: Dietician  **Diet Quality type**: NR | **Energy including alcohol:** 1923±382 kcal/day  **Energy excluding alcohol:** NR  **Alcohol (g):** NR | **AUD:**  NR | **AUD:**  NR | **AUD:**  NR | Participants with cirrhosis had lower body weight as compared to healthy controls and were taking in under 2000 kcal a day including alcohol, suggesting participants were not taking in a normal nutritional intake. |
| **Reference** | **Study sample information** | **Study design and history of alcohol use** | **Demographics** | **Dietary assessment information** | **Energy and alcohol intake** | **Macronutrients**  **Carbohydrate Protein Fat** | | | **Summary and dietary intake findings**  Were participants taking in normal amount of food and macronutrients? |
| Chang et al., 2003 | **Dietary Period of Assessment during Study:** Real-world  **AUD Population** **Sample Size:** Total: 24 M: 24 F: 0  **Diet as Primary Outcome:** No | **Study Design:** Cross Sectional  **Drinking status**: Active   **Diagnosis Type:**  Alcohol- induced Liver cirrhosis  **Patient Type:** Physiological Diagnosis of AUD | **Race/Ethnicity:**  Korean  **Age**: 54.8±9.20  **BMI**: 23.7±3.22 | **Diet tool used**: Diet recall and Food record/diary  **Timeframe of assessment**: 24-hr recall  **Diet assessment conductor**: Dietician  **Diet Quality type**: NR | **Energy including alcohol:** 1682±410 kcal/day  **Energy excluding alcohol:** 1448±483 kcal/day  **Alcohol (g):** NR | **AUD:**  256±64 g/day | **AUD:**  58±20 g/day | **AUD:**  31±13 g/day | Participants appeared to be consuming under 2000 kcal/day both when alcohol is and is not accounted for. Participants body fat was also reported to be impacted by the severity of their liver cirrhosis. |
| Cunha et al., 2004 | **Dietary Period of Assessment during Study:** Real-world  **AUD Population** **Sample Size:** Total: 29 M: 21 F: 8  **Diet as Primary Outcome:** Yes | **Study Design:** Longitudinal  **Drinking status**: Active   **Diagnosis Type:**  Alcohol- associated Liver cirrhosis  **Patient Type:** Physiological Diagnosis of AUD | **Race/Ethnicity:**  N/R  **Age**: 52  **Weight:**62±10 kg | **Diet tool used**: Unclear  **Timeframe of assessment**: 3- day recall  **Diet assessment conductor**: dietician  **Diet Quality type**: NR | **Energy including alcohol:** 2327±857 kcal/day  **Energy excluding alcohol:** 1873±679 kcal/day  **Alcohol (g):** NR | **AUD:**  238±92 g/day | **AUD:**  65±25 g/day | **AUD:**  74±33 g/day | Prior to nutrition counseling, participants appear to be consuming slightly under 2000 kcal/day without alcohol. Nutritional counseling appeared to significantly improve participants nutritional status. |
| **Reference** | **Study sample information** | **Study design and history of alcohol use** | **Demographics** | **Dietary assessment information** | **Energy and alcohol intake** | **Macronutrients**  **Carbohydrate Protein Fat** | | | **Summary and dietary intake findings**  Were participants taking in normal amount of food and macronutrients? |
| De Timary et al., 2012 | **Dietary Period of Assessment during Study:** Real-world  **AUD Population Sample Size:** Total: 97 M: 63 F: 34  **Diet as Primary Outcome:** No | **Study Design:** Cross-Sectional  **Drinking status**:  Active   **Diagnosis Type:** Alcohol dependence  (DSM-IV)  **Patient Type:** Clinical Diagnosis of AUD | **Race/Ethnicity:**  N/R  **Age**: 49±11  **BMI**: 24.7±4.5 | **Diet tool used**: Diet history interview  **Timeframe of assessment**: 7-day recall  **Diet assessment conductor**: Dietician  **Diet Quality type**: NR | **Energy including alcohol:** 39.0±19.1 kcal/kg/day  **Energy excluding alcohol:** 23.5±16.0 kcal/kg/day  **Alcohol(g):** 159.4±108.7 | **AUD:**  31.6±9.7% | **AUD:**  9.8±3.7% | **AUD:**  19.5±8.3% | As alcohol consumption increased, consumption of other kcal initially appeared to decrease and then plateaued, meaning that those consuming the most alcohol consumed far above the norm when alcohol intake is included. |
| Delarue et al., 1996 | **Dietary Period of Assessment during Study:** Real-world    **AUD Population Sample Size:** Total: 24 M: 24 F: 0  **Diet as Primary Outcome:** No | **Study Design:** Cross-Sectional  **Drinking status**: Active   **Diagnosis Type:** Alcohol dependence  (Not specified)  **Patient Type:** Other- participants are referred as ‘alcoholic’ but it is not clarified how they were diagnosed | **Race/Ethnicity:**  N/R  **Age**: 39.7  **BMI**: 22.8±3.7 | **Diet tool used**: Diet recall  **Timeframe of assessment**: 7-day recall  **Diet assessment conductor**: NR  **Diet Quality type**: NR | **Energy including alcohol:** 2390.06 kcal/day  **Energy excluding alcohol:** NR  **Alcohol (g):**185±90 | **AUD:**  291±138 g/day | **AUD:**  92±24 g/day | **AUD:**  94±39 g/day | Participants carbohydrate intake increased after alcohol withdrawal. |
| **Reference** | **Study sample information** | **Study design and history of alcohol use** | **Demographics** | **Dietary assessment information** | **Energy and alcohol intake** | **Macronutrients**  **Carbohydrate Protein Fat** | | | **Summary and dietary intake findings**  Were participants taking in normal amount of food and macronutrients? |
| Gloria et al., 1997 | **Dietary Period of Assessment during Study:** Real-world    **AUD Population Sample Size:** Total: 33 M: 23 F: 10  **Diet as Primary Outcome:** Yes | **Study Design:** Descriptive  **Drinking status**: Active   **Diagnosis Type:** Alcohol dependence  (DSM-III)  **Patient Type:** Clinical Diagnosis of AUD | **Race/Ethnicity:**  White/ Caucasian  **Age**: 42  **BMI**:23.8±2.4 | **Diet tool used**: food frequency questionnaire  **Timeframe of assessment:** Diet history  **Diet assessment conductor**: dietician  **Diet Quality type**: NR | **Energy including alcohol:** 173.4±44.6 kJ/kg/day  **Energy excluding alcohol:** 96.5±31.7 kJ/kg/day  **Alcohol:** 79±43 g/kg/day | **AUD:**  2.6 g/kg/day | **AUD:**  1.08 g/kg/day | **AUD:**  0.94 g/kg/day | The majority of participants were not malnourished and were consuming normal amounts of food, but an inverse correlation was observed between alcohol intake and weight-height index. |
| Hurt et al., 1981 | **Dietary Period of Assessment during Study:** Real-world  **AUD Population** **Sample Size:** Total: 58 M: 43 F: 15  **Diet as Primary Outcome:** Yes | **Study Design:** Longitudinal  **Drinking status**: Active   **Diagnosis Type:** Alcohol dependence  (Based on physician diagnosis, not otherwise specified)  **Patient Type:** Clinical Diagnosis of AUD | **Race/Ethnicity:**  N/R  **Age**: 44.5±12.2  **Weight:** 68.2±13.95 kg | **Diet tool used**: Diet History interview  **Timeframe of assessment**: Diet history  **Diet assessment conductor**: Dietician  **Diet Quality type**: NR | **Energy including alcohol:** NR for whole AUD population (2598 kcal/day recorded for AUD population subset)  **Energy excluding alcohol:** 2029±751 kcal/day  **Alcohol:**161±95.67 g | **AUD:**  161±95.67 g/day | **AUD:**  86±32g/day | **AUD:**  105±45g/day | The vast majority of participants consumed normal amounts of food and macronutrients. |
| **Reference** | **Study sample information** | **Study design and history of alcohol use** | **Demographics** | **Dietary assessment information** | **Energy and alcohol intake** | **Macronutrients**  **Carbohydrate Protein Fat** | | | **Summary and dietary intake findings**  Were participants taking in normal amount of food and macronutrients? |
| Laitinen et al., 1990 | **Dietary Period of Assessment during Study:** Real-world  **AUD Population** **Sample Size:** Total: 38 M: 38 F: 0  **Diet as Primary Outcome:** No | **Study Design:** Cross- sectional  **Drinking status**: Active   **Diagnosis Type:** Alcohol dependence  (not specified)  **Patient Type:** participants are referred as ‘alcoholic’ but it is not clarified how they were diagnosed | **Race/Ethnicity:**  Finnish  **Age**: 37  **Weight:** 73.5 kg | **Diet tool used**: Diet History interview  **Timeframe of assessment**: Monthly recall  **Diet assessment conductor**: Nutritionist  **Diet Quality type**: NR | **Energy including alcohol:** 3030 kcal/day  **Energy excluding alcohol:** NR  **Alcohol (g):**131±63 | **AUD:**  266±56 g/day | **AUD:**  82±20 g/day | **AUD:**  100±30 g/day | Participants with AUD consumed slightly less protein and slightly more carbohydrates and fat as compared to the control group. |
| Lévy et al., 1995 | **Dietary Period of Assessment during Study:** Real-world    **AUD Population Sample Size:** Total: 106 M: 106 F: 0  **Diet as Primary Outcome:** No | **Study Design**: Case-Control  **Drinking status**: Active   **Diagnosis Type:**  Alcohol-associated liver cirrhosis and pancreatitis  **Patient Type:** Physiological Diagnosis of AUD | **Race/Ethnicity:**  N/R  **Age**: 46.9±9.6  **Weight:**68.7 ±11.5 kg | **Diet tool used**: Diet History interview  **Timeframe of assessment**: Diet history  **Diet assessment conductor**: Nutritionist  **Diet Quality type**: NR | **Energy including alcohol:** 410.4 kcal/wk/kg  **Energy excluding alcohol:** 298 kcal/wk/kg  **Alcohol:** 27.6% | **AUD:**  29.3% | **AUD:**  11% | **AUD:**  32.2% | Participants with alcohol-associated chronic pancreatitis consumed more non-alcohol energy as compared to participants with alcohol-associated cirrhosis. |
| **Reference** | **Study sample information** | **Study design and history of alcohol use** | **Demographics** | **Dietary assessment information** | **Energy and alcohol intake** | **Macronutrients**  **Carbohydrate Protein Fat** | | | **Summary and dietary intake findings**  Were participants taking in normal amount of food and macronutrients? |
| Manari et al., 2003 | **Dietary Period of Assessment during Study:** Real-world  **AUD Population** **Sample Size:** Total: 30 M: 27 F: 3  **Diet as Primary Outcome:** Yes | **Study Design:** Cross Sectional  **Drinking status**: Active  **Diagnosis Type:** Alcohol dependence  (SADQ and AUDIT)  **Patient Type:** Clinical Diagnosis of AUD | **Race/Ethnicity:**  White English, Celtic, Other  **Age**: 49±2.4  **BMI**: 22.6±1.2 | **Diet tool used**: Diet recall  **Timeframe of assessment**: 24 hr recall  **Diet assessment conductor**: NR  **Diet Quality type**: Recommended Nutrient Intake (based on United Kingdom guidelines | **Energy including alcohol:** 2939.77 kcal/day  **Energy excluding alcohol:** 1441.20 kcal/day  **Alcohol (g):** 162±15 | **AUD:**  237±32 g/day | **AUD:**  103±14 g/day | **AUD:**  55.5±5.2 g/day | Participants appeared to consume roughly average energy, but the paper reported concerns regarding participants ability to accurately recall their diet due to their alcohol drinking. |
| Manocha et al., 1989 | **Dietary Period of Assessment during Study:** Real-world  **AUD Population Sample Size:** Total: 11 M: 11 F: 0  **Diet as Primary Outcome:** Yes | **Study Design:** Cross sectional  **Drinking status**: Active   **Diagnosis Type:**  Alcohol- induced Liver cirrhosis  **Patient Type:** Physiological Diagnosis of AUD | **Race/Ethnicity:**  South Asian Indian  **Age**: 43 ±11  **Weight:** 61.9 kg | **Diet tool used**: Diet history interview  **Timeframe of assessment**: 24 hr recall  **Diet assessment conductor**: NR  **Diet Quality type**: NR | **Energy including alcohol:** 1541±428 kcal/day  **Energy excluding alcohol:** NR  **Alcohol (g):** NR | **AUD:**  241±63 g | **AUD:**  52±16 g | **AUD:**  41±15g | There were no significant differences in the nutritional intake of the patients with alcohol-associated cirrhosis and the healthy controls. |
| **Reference** | **Study sample information** | **Study design and history of alcohol use** | **Demographics** | **Dietary assessment information** | **Energy and alcohol intake** | **Macronutrients**  **Carbohydrate Protein Fat** | | | **Summary and dietary intake findings**  Were participants taking in normal amount of food and macronutrients? |
| Melgaard et al., 1989 | **Dietary Period of Assessment during Study:** Real-world  **AUD Population**  **Sample Size:** Total: 45 M: 45 F: 0  **Diet as Primary Outcome:** Yes | **Study Design:** Cross-Sectional  **Drinking status**: Active   **Diagnosis Type:**  Alcohol dependence (MASS)  **Patient Type:** Clinical Diagnosis of AUD | **Race/Ethnicity:**  N/R  **Age**: 43  **BMI/Weight:** No data | **Diet tool used:** Diet history interview  **Timeframe of assessment**: Diet history  **Diet assessment conductor**: Dietician  **Diet Quality type**: NR | **Energy including alcohol:** 2072.18 kcal/day  **Energy excluding alcohol:** NR  **Alcohol (g)**:NR | **AUD:**  NR | **AUD:**  72±32 g/day | **AUD:**  110±57 g/day | Participants were not generally malnourished but did frequently have a folate deficit. |
| Mendenhall et al., 1985 | **Dietary Period of Assessment during Study:** Real-world  **AUD Population**  **Sample Size:** Total: 52 M: no data F: no data  **Diet as Primary Outcome:** Yes | **Study Design:** Experimental  **Drinking status**: Active   **Diagnosis Type:**  Alcohol- induced Liver hepatitis  **Patient Type:** Physiological Diagnosis of AUD | **Race/Ethnicity:**  **N/R**  **Age**: 47.8±1.8  **BMI/Weight**: no data | **Diet tool used:** Diet history interview  **Timeframe of assessment**: monthly recall  **Diet assessment conductor**: NR  **Diet Quality type**: NR | **Energy including alcohol:** 5193.4 ±260 kcal/day  **Energy excluding alcohol:** 3422.6±288.9 kcal/day  **Alcohol (g):**255.5±33.4 | **AUD:**  NR | **AUD:**  55±5.4 g/day | **AUD:**  NR | Participants were consuming on average, over 3000 kcal/day, but on average were consuming about 1700 kcal/day from alcohol, suggesting that they were not consuming a normal amount of food.  This study highlighted the need for careful nutritional  assessments in this patient population and nutritional  supplementation during the anorectic phase of their disease.  The long-term effects of restoring nutritional status  in terms of recovery, relapses, associated complications,  and progression of the liver disease requires additional  long-term observations. |
| **Reference** | **Study sample information** | **Study design and history of alcohol use** | **Demographics** | **Dietary assessment information** | **Energy and alcohol intake** | **Macronutrients**  **Carbohydrate Protein Fat** | | | **Summary and dietary intake findings**  Were participants taking in normal amount of food and macronutrients? |
| Mendenhall et al., 1993 | **Dietary Period of Assessment during Study:** Real-world  **AUD Population** **Sample Size:** Total: 273 M: 273 F: 0  **Diet as Primary Outcome:** Yes | **Study Design:** Experimental  **Drinking status**: Active   **Diagnosis Type:**  Alcohol- associated liver hepatitis  **Patient Type:** Physiological Diagnosis of AUD | **Race/Ethnicity:**  **N/R**  **Age**: 50.9±9.6  **BMI**: no data  **Ideal body weight (%):** 100% | **Diet tool used:** Diet history interview  **Timeframe of assessment**: monthly recall  **Diet assessment conductor**: Dietician and self  **Diet Quality type**: NR | **Energy including alcohol:** 2754±1506.3 kcal/day  **Energy excluding alcohol:** NR  **Alcohol (g):**206.1±176.3 | **AUD:**  NR | **AUD:**  NR | **AUD:**  NR | Nutritional status across all participants was reported to be abnormal. . |
| Mezey et al., 1988 | **Dietary Period of Assessment during Study:** Real-world  **AUD Population** **Sample Size:** Total: 129 M: 80 F: 49  **Diet as Primary Outcome:** Yes | **Study Design**: Cross- Sectional  **Drinking status**: Active   **Diagnosis Type:**  Alcohol-associated liver cirrhosis and pancreatitis  **Patient Type:** Physiological Diagnosis of AUD | **Race/Ethnicity:**  **N/R**  **Age**: 42.4±1.7  **Weight % of Standard**: 99.4±3.3 | **Diet tool used:** Diet history interview  **Timeframe of assessment**: Diet history  **Diet assessment conductor**: Dietician  **Diet Quality type**: NR | **Energy including alcohol:** 2520.1±130.8 kcal/day  **Energy excluding alcohol:** 1256.4±95.9 kcal/day  **Alcohol (g):**181.4±15.4 | **AUD:**  21.6±1.5% | **AUD:**  8.4±0.6% | **AUD:**  19.9±1.5% | Participants were ingesting roughly half of daily calories from alcohol, and had low mean intake of protein, fat, and carbohydrates. |
| **Reference** | **Study sample information** | **Study design and history of alcohol use** | **Demographics** | **Dietary assessment information** | **Energy and alcohol intake** | **Macronutrients**  **Carbohydrate Protein Fat** | | | **Summary and dietary intake findings**  Were participants taking in normal amount of food and macronutrients? |
| Mills et al., 1983 | **Dietary Period of Assessment during Study:** Real-world  **AUD Population** **Sample Size:** Total: 30 M: 22 F: 8  **Diet as Primary Outcome:** No | **Study Design:** Longitudinal  **Drinking status**: Active   **Diagnosis Type:**  Alcohol-associated liver Disease  **Patient Type:** Physiological Diagnosis of AUD | **Race/Ethnicity:**  **N/R**  **Age**: 48.6  **Weight % of Standard:** 107.8% | **Diet tool used:** Diet history interview  **Timeframe of assessment**: 6 month recall  **Diet assessment conductor**: Dietician  **Diet Quality type**: NR | **Energy including alcohol:** 2452±225 kcal/day  **Energy excluding alcohol:** NR  **Alcohol (g):** 165 g | **AUD:**  NR | **AUD:**  58.5±5.4 g/day | **AUD:**  NR | 22% of participants were grossly deficient in protein, but mean daily energy was adequate. |
| Neville et al., 1968 | **Dietary Period of Assessment during Study:** Real-world  **AUD Population** **Sample Size:** Total: 34 M: 26 F: 8  **Diet as Primary Outcome:** Yes | **Study Design:** Cross- Sectional  **Drinking status**: Active   **Diagnosis Type:**  Alcohol dependence (not specified) and alcohol-associated liver disease  **Patient Type:** Clinical/Physiological Diagnosis of AUD | **Race/Ethnicity:**  White, Other  **Age**: 42.32  **Weight**: 142.12 lb | **Diet tool used:** Diet history interview  **Timeframe of assessment**: Diet history  **Diet assessment conductor**: NR  **Diet Quality type**: NR | **Energy including alcohol:** 2678.94±1056.76 kcal/day  **Energy excluding alcohol:** NR  **Alcohol (g):**126.9±91.12 | **AUD:**  33.0% | **AUD:**  10.3% | **AUD:**  23.8% | Participants appeared to have roughly adequate intake once they were given diet therapy and vitamins. Before that, they were severely malnourished. |
| **Reference** | **Study sample information** | **Study design and history of alcohol use** | **Demographics** | **Dietary assessment information** | **Energy and alcohol intake** | **Macronutrients**  **Carbohydrate Protein Fat** | | | **Summary and dietary intake findings**  Were participants taking in normal amount of food and macronutrients? |
| NICOLÁS et al., 1993 | **Dietary Period of Assessment during Study:** Real-world  **AUD Population Sample Size:** Total: 250 M: 250 F: 0  **Diet as Primary Outcome:** Yes | **Study Design:** Cross-Sectional  **Drinking status**: Active   **Diagnosis Type:**  Alcohol dependence- DSM-III-R  **Patient Type:** Clinical Diagnosis of AUD | **Race/Ethnicity:**  White**/**Caucasian  **Age**: 41±11  **Weight % of standard:**  99.5±16.3 | **Diet tool used:** Retrospective structured questionnaire  **Timeframe of assessment**: monthly recall  **Diet assessment conductor**: physician and self  **Diet Quality type**: NR | **Energy including alcohol:** 3250±622 kcal/day  **Energy excluding alcohol:** 1620±450 kcal/day  **Alcohol (g):**235±62 | **AUD:**  NR | **AUD:**  NR | **AUD:**  NR | No, participants with AUD were consuming markedly less than the ideal 2000 kcal/day, and significantly fewer kcal/day than the healthy controls |
| Nielsen et al., 1993 | **Dietary Period of Assessment during Study:** Inpatient  **AUD Population**  **Sample Size:** Total: 37  M: 26 F: 11  **Diet as Primary Outcome:** Yes | **Study Design:** Cross-Sectional  **Drinking status**: Abstinent   **Diagnosis Type:**  Alcohol-associated liver cirrhosis  **Patient Type:** Physiological Diagnosis of AUD | **Race/Ethnicity:**  **N/R**  **Age**: 46  **Weight:** 59.7 kg | **Diet tool used**: Diet recall  **Timeframe of assessment**: NR  **Diet assessment conductor**: dietician  **Diet Quality type**: NR | **Energy including alcohol:** 1720.84 kcal/day  **Energy excluding alcohol:** NR  **Alcohol (g):**100 | **AUD:**  NR | **AUD:**  54g/day | **AUD:**  NR | Over half of the participants were malnourished, so they were not consuming adequate nutrition. |
| **Reference** | **Study sample information** | **Study design and history of alcohol use** | **Demographics** | **Dietary assessment information** | **Energy and alcohol intake** | **Macronutrients**  **Carbohydrate Protein Fat** | | | **Summary and dietary intake findings**  Were participants taking in normal amount of food and macronutrients? |
| NOEL-JORAND & BRAS, 1994 | **Dietary Period of Assessment during Study:** Real-world  **AUD Population Sample Size:** Total: 72 M: 59 F: 13  **Diet as Primary Outcome:** No | **Study Design: Multifactorial**  **Drinking status**: Active   **Diagnosis Type:**  Alcohol-associated liver cirrhosis and pancreatitis  **Patient Type:** Physiological Diagnosis of AUD | **Race/Ethnicity:**  N/R  **Age**: 44.38±8.11  **BMI**: 22.7±3.8 | **Diet tool used:** Retrospective structured questionnaire  **Timeframe of assessment**: Diet history  **Diet assessment conductor**: Trained Professional  **Diet Quality type**: NR | **Energy including alcohol:** 4376 kcal/day  **Energy excluding alcohol:** 2842 kcal/day  **Alcohol (g):**210 | **AUD:**  421 g/day | **AUD:**  114.4 g/day | **AUD:**  110.0 g/day | Participants and controls did not differ on protein intake, but seemed to consume more fat than controls, more carbohydrates, and more energy overall when including alcohol. |
| Palliyath & Schwartz, 1993 | **Dietary Period of Assessment during Study:** Real-world  **AUD Population Sample Size:** Total: 24 M: no data F: no data  **Diet as Primary Outcome:** No | **Study Design:** Longitudinal  **Drinking status**: Active  **Diagnosis Type:**  Alcohol dependence (not specified)  **Patient Type:** Clinical Diagnosis of AUD | **Race/Ethnicity:**  N/R **Age**: 46 ±10  **Weight:** 163±27 lb | **Diet tool used**: Diet history interview  **Timeframe of assessment**: Diet history  **Diet assessment conductor:** dietician  **Diet Quality type**: NR | **Energy including alcohol:** 1850±450 kcal/day  **Energy excluding alcohol:** NR  **Alcohol (g):** 207±25 | **AUD:**  NR | **AUD:**  64±8 g/day | **AUD:**  NR | Participants reported having adequate nutritional status.  Participants did not have a significant change in diet that caused any direct impact to their overall health. |
| **Reference** | **Study sample information** | **Study design and history of alcohol use** | **Demographics** | **Dietary assessment information** | **Energy and alcohol intake** | **Macronutrients**  **Carbohydrate Protein Fat** | | | **Summary and dietary intake findings**  Were participants taking in normal amount of food and macronutrients? |
| Panagaria et al., 2007 | **Dietary Period of Assessment during Study:** Real-world  **AUD Population Sample Size:** Total:66 M: 66 F: 0  **Diet as Primary Outcome:** Yes | **Study Design:** Prospective Longitudinal  **Drinking status**: Active   **Diagnosis Type:**  Alcohol-associated liver disease and alcohol dependence (not specified)  **Patient Type:** Clinical/ Physiological Diagnosis of AUD | **Race/Ethnicity:**  South Asian Indian  **Age**: 40.15±6.96  **BMI**:21.84±3.16 | **Diet tool used**: Diet recall  **Timeframe of assessment**: 48 hr- recall  **Diet assessment conductor**: NR  **Diet Quality type**: NR | **Energy including alcohol:** 983.66±313.59 kcal/day  **Energy excluding alcohol:** NR  **Alcohol (g):**161.64±126.88 | **AUD:**  174.7±85.69g/day | **AUD:**  38.5±22.33g/day | **AUD:**  32.89±23.22 g/day | All participants with alcohol-associated liver disease qualified as malnourished, and 60% of participants with alcohol abuse qualified as malnourished. |
| Pezzarossa et al., 1986 | **Dietary Period of Assessment during Study:** Real-world  **AUD Population Sample Size:** Total: 22 M: 16 F: 6  **Diet as Primary Outcome:** No | **Study Design:** Longitudinal  **Drinking status**: Active   **Diagnosis Type:** Alcohol dependence  (Not specified)  **Patient Type:** Other- Unclear, but the article reports that participants had been ingesting more than 200 g/day of alcohol | **Race/Ethnicity:**  NR  **Age**: 37.77±2.64  **Weight**:66.05±3.09 kg | **Diet tool used**: Diet History interview  **Timeframe of assessment**: Diet history  **Diet assessment conductor**: NR  **Diet Quality type**: NR | **Energy including alcohol:** 2805±91 kcal/day  **Energy excluding alcohol:** 1165.91±87.73 kcal/day  **Alcohol (g):**234.09±15.18 | **AUD:**  NR | **AUD:**  NR | **AUD:**  NR | Participant diets were 58% alcohol by caloric value; they were significantly under consuming food and all macronutrients. This was shown through the lack of recovery in withdrawal because of their malnutrition. |
| **Reference** | **Study sample information** | **Study design and history of alcohol use** | **Demographics** | **Dietary assessment information** | **Energy and alcohol intake** | **Macronutrients**  **Carbohydrate Protein Fat** | | | **Summary and dietary intake findings**  Were participants taking in normal amount of food and macronutrients? |
| Pitchumoni et al., 1980 | **Dietary Period of Assessment during Study:** Real-world  **Sample Size:** Total: 62 M: no data F: no data  **Diet as Primary Outcome:** Yes | **Study Design:** Cross-sectional  **Drinking status**: Active    **Diagnosis Type:**  Alcohol-associated liver Cirrhosis and pancreatitis  **Patient Type:** Physiological Diagnosis of AUD | **Race/Ethnicity:**  N/R  **Age**: NR  **BMI**: NR | **Diet tool used**: Diet History interview  **Timeframe of assessment**: Diet history  **Diet assessment conductor**: Self  **Diet Quality type**: NR | **Energy including alcohol:** NR  **Energy excluding alcohol:** 949.35±442.06 kcal/day  **Alcohol (g):** 201 g/day (paper states participants consumed >200 g/day) | **AUD:**  NR | **AUD:**  62.4±22.42 g/day | **AUD:**  51.0±28.39 g/ day | Participants with alcohol-associated cirrhosis had worse nutrition as compared to those with pancreatitis, but participants with pancreatitis also consume less nutrients than the national average. |
| **Reference** | **Study sample information** | **Study design and history of alcohol use** | **Demographics** | **Dietary assessment information** | **Energy and alcohol intake** | **Macronutrients**  **Carbohydrate Protein Fat** | | | **Summary and dietary intake findings**  Were participants taking in normal amount of food and macronutrients? |
| Ratteree et al., 2019 | **Dietary Period of Assessment during Study:** Real-world  **AUD Population**  **Sample Size:** Total: 21 M: 14 F: 7  **Diet as Primary Outcome:** Yes | **Study Design**: Observational  **Drinking status**: Active  **Diagnosis Type:** AUD- (DSM-5)  **Patient Type:** Clinical Diagnosis of AUD | **Race/Ethnicity:**  White, African American, Multiracial, Unknown  **Age**: 46.3±2.8  **BMI**:23.8±0.6 | **Diet tool used**: Food Frequency Questionnaire  **Timeframe of assessment**: 1 year recall  **Diet assessment conductor**: Dietician  **Diet Quality type**: NR | **Energy including alcohol:** 6683.01 kcal/day  **Energy excluding alcohol:** 3372.20 kcal/day  **Alcohol (g):**472.96±75.8 | **AUD:**  514.3±66.8 g/day | **AUD:**  122.8±17.0 g/day | **AUD:**  101.2±13.6 g/day | This is difficult to know as this paper casts doubt generally on the ability of participants with AUD to accurately recall their diet but is does say that as about 45% of calories in participants with AUD diets came from alcohol, this did affect the ratio at which they consumed other macronutrients. |
| Rintamäki et al., 2014 | **Dietary Period of Assessment during Study:** Real-world  **AUD Population**  **Sample Size:** Total: 200 M: 167 F: 33  **Diet as Primary Outcome:** No | **Study Design:** Cross-Sectional  **Drinking status**: Active   **Diagnosis Type:** AUD (M-CIDI)  **Patient Type:** Clinical Diagnosis of AUD | **Race/Ethnicity:**  NR  **Age**: 45.94±10.58  **BMI**:27.02±4.13 | **Diet tool used**:  Food Frequency Questionnaire  **Timeframe of assessment**: 1- year recall  **Diet assessment conductor**: Self  **Diet Quality type**: NR | **Energy including alcohol:** 2301.63 kcal/day  **Energy excluding alcohol:** NR  **Alcohol:** 4.21% | **AUD:**  40.83% | **AUD:**  17.17% | **AUD:**  37.34% | Participants with AUD consumed fewer carbohydrates than other groups, and women with AUD consumed less sucrose and more fats, while men with AUD consumed much less fiber than other groups. |
| **Reference** | **Study sample information** | **Study design and history of alcohol use** | **Demographics** | **Dietary assessment information** | **Energy and alcohol intake** | **Macronutrients**  **Carbohydrate Protein Fat** | | | **Summary and dietary intake findings**  Were participants taking in normal amount of food and macronutrients? |
| Sangwan & Khetarpaul, 1998 | **Dietary Period of Assessment during Study:** Real-world  **AUD Population Sample Size:** Total: 60 M: 60 F: 0  **Diet as Primary Outcome:** Yes | **Study Design:** Cross-Sectional  **Drinking status**: Active  **Diagnosis Type:** Alcohol dependence  (Not specified)  **Patient Type:** Other- Unclear with a minimum amount of alcohol consumption listed for all participants 400 ml/day | **Race/Ethnicity:**  South Asian Indian  **Age**: NR  **BMI**: NR | **Diet tool used**: Diet recall  **Timeframe of assessment**: 24 hrrecall  **Diet assessment conductor**: NR  **Diet Quality type**: NR | **Energy including alcohol:** 3694±985.4 kcal/day  **Energy excluding alcohol:** 2060±395.0 kcal/day  **Alcohol (g):** NR | **AUD:**  266±77.8 g/day | **AUD:**  84±25.40 g/day | **AUD:**  32±5.3 g/day | Participants consumed up to 44% alcohol by energy, and participants consumed 20-73% less vegetables, cereals, sugar, and fats and oils than recommended. |
| Sarin et al., 1997 | **Dietary Period of Assessment during Study:** Real-world  **AUD Population Sample Size:** Total: 119 M: no data F: no data  **Diet as Primary Outcome:** Yes | **Study Design:** Prospective  **Drinking status**: Active   **Diagnosis Type:**  Alcohol- associated liver Disease and alcohol dependence (unspecified)  **Patient Type:** Physiological/ Clinical Diagnosis of AUD | **Race/Ethnicity:**  NR  **Age**:40.8±8.79  **Weight:** 60.3±10.56 kg | **Diet tool used**: Unclear  **Timeframe of assessment**: 72 hr -recall  **Diet assessment conductor**: dietician  **Diet Quality type**: NR | **Energy including alcohol:** 2834.64 kcal/day  **Energy excluding alcohol:**  1481.45 ±493.56 kcal/day  **Alcohol (g):**190.1±134.71 | **AUD:**  172.5±75 g/day | **AUD:**  49±20.43 g/day | **AUD:**  47.4±17.47 g/ day | Both AUD and ALD participants had significant dietary disturbances, and similar risk of malnutrition. |
| **Reference** | **Study sample information** | **Study design and history of alcohol use** | **Demographics** | **Dietary assessment information** | **Energy and alcohol intake** | **Macronutrients**  **Carbohydrate Protein Fat** | | | **Summary and dietary intake findings**  Were participants taking in normal amount of food and macronutrients? |
| Simko et al., 1982 | **Dietary Period of Assessment during Study:** Real-world  **AUD Population**  **Sample Size:** Total: 82 M: 75 F: 8  **Diet as Primary Outcome:** Yes | **Study type:** Experimental  **Drinking status**: Active   **Diagnosis Type:**  Alcohol-associated liver Disease and alcohol dependence (unspecified)  **Patient Type:** Physiological/ Clinical Diagnosis of AUD | **Race/Ethnicity:**  Caucasian, Black  **Age**:51.3±1.62  **Weight**: 70.9±2.20 kg | **Diet tool used**: Diet history interview  **Timeframe of assessment**: Diet history  **Diet assessment conductor**: nutritionist  **Diet Quality type**: NR | **Energy including alcohol:** 29.86±2.04 kcal/kg/day  **Energy excluding alcohol:** NR  **Alcohol:**1.99±0.17 g/kg/day | **AUD:**  NR | **AUD:**  0.95±0.06 g/kg/day | **AUD:**  NR | In comparison to the control group the group with ALD and the AUD group had both lower weight and a lower weight to height index. These two groups also gained a substantial amount of their caloric intake form alcohol each day. |
| Sobral-Oliveira et al., 2011 | **Dietary Period of Assessment during Study:** Real-world  **AUD Population Sample Size:** Total: 32 M: 32 F: 0  **Diet as Primary Outcome:** No | **Study Design:** Observational  **Drinking status**: Active  **Diagnosis Type:**  Alcohol- associated pancreatitis and alcohol dependence (CARET)  **Patient Type:** Physiological/ Clinical Diagnosis of AUD | **Race/Ethnicity:**  **N/R**  **Age**: 54.5±10.3  **BMI**:24.6±5.4 | **Diet tool used**: Diet recall  **Timeframe of assessment**: 7 day recall  **Diet assessment conductor**: dietician  **Diet Quality type**: NR | **Energy including alcohol:** NR  **Energy excluding alcohol:** 1640±457.5 kcal/day  **Alcohol (g):**311.0±213.5 g/day | **AUD:**  231.5±73.1 g/day | **AUD:**  67.6±20.3 g/day | **AUD:**  49.9±15.9 g/day | General diet composition was found to be acceptable across all participants. |
| **Reference** | **Study sample information** | **Study design and history of alcohol use** | **Demographics** | **Dietary assessment information** | **Energy and alcohol intake** | **Macronutrients**  **Carbohydrate Protein Fat** | | | **Summary and dietary intake findings**  Were participants taking in normal amount of food and macronutrients? |
| Videla et al., 1984 | **Dietary Period of Assessment during Study:** Real-world  **AUD Population Sample Size:** Total: 24 M: 24 F: 0  **Diet as Primary Outcome:** No | **Study Design:** Cross Sectional  **Drinking status**: Active   **Diagnosis Type:**  Alcohol dependence (not specified)  **Patient Type:** Clinical Diagnosis of AUD | **Race/Ethnicity:**  **N/R Age**: 43±6.9  **Weight % of Standard:** 99.6±3.7 | **Diet tool used**: Diet recall  **Timeframe of assessment**: 24 hr recall  **Diet assessment conductor**: NR  **Diet Quality type**: NR | **Energy including alcohol:** 46.7 ±3.7 ideal weight/kcal/kg  **Energy excluding alcohol:** 13.8±3.8 ideal weight/kcal/kg  **Alcohol (g):** NR | **AUD:**  NR | **AUD:**  0.51±0.14 ideal weight/g/kg | **AUD:**  NR | There were no significant differences in the diets of the groups in the study. |
| Wagnerberger et al., 2007 | **Dietary Period of Assessment during Study:** Real-world  **AUD Population Sample Size:** Total: 210 M: 158 F: 52  **Diet as Primary Outcome:** Yes | **Study Design:** Cross-Sectional  **Drinking status**: Active   **Diagnosis Type:**  Alcohol-associated liver Disease  **Patient Type:** Physiological Diagnosis of AUD | **Race/Ethnicity:**  NR  **Age**: 46.4±1.4  **BMI**:24.3±0.7 | **Diet tool used**: Diet History interview  **Timeframe of assessment**: Diet history  **Diet assessment conductor**: nutritionist  **Diet Quality type**: NR | **Energy including alcohol:** 3311.8±185.3 kcal/day  **Energy excluding alcohol:** 2402.8±148.9 kcal/day  **Alcohol (g):**128±12.0 | **AUD:**  287.4±20.3 g/day | **AUD:**  86.5 ±6.0 g/day | **AUD:**  93.4±7.1 g/day | After excluding energy intake from alcohol, the study reported no significant differences in energy or macronutrient intake between the AUD groups and the healthy controls. |
| **Reference** | **Study sample information** | **Study design and history of alcohol use** | **Demographics** | **Dietary assessment information** | **Energy and alcohol intake** | **Macronutrients**  **Carbohydrate Protein Fat** | | | **Summary and dietary intake findings**  Were participants taking in normal amount of food and macronutrients? |
| Wilkens Knudsen et al., 2014 | **Dietary Period of Assessment during Study:** Real-world  **AUD Population**  **Sample Size:** Total: 49 M: 30 F: 19  **Diet as Primary Outcome:** Yes | **Study Design:** Cross Sectional  **Drinking status**: Abstinent   **Diagnosis Type:**  Alcohol dependence (ICD-10)  **Patient Type:** Clinical Diagnosis of AUD | **Race/Ethnicity:**  Danish  **Age**: 49  **BMI**:25 | **Diet tool used**: Food record/diary  **Timeframe of assessment**: 7-day recall  **Diet assessment conductor**: dietician  **Diet Quality type**: NR | **Energy including alcohol:** 2007.89 kcal/day  **Energy excluding alcohol:** NR  **Alcohol (g):**171.0 | **AUD:**  206 g | **AUD:**  82 g | **AUD:**  82 g | Overall, the energy intake of the AUD participants was lower than the general population, and participants were more likely to develop micronutrient deficits. |
| Wilson et al., 1985 | **Dietary Period of Assessment during Study:** Real-world  **Sample Size:** Total: 53 M: NR F: NR  **Diet as Primary Outcome:** Yes | **Study Design: Cross-sectional**  **Drinking status**: Active  **Diagnosis Type:**  Alcohol-associated -pancreatitis and liver Disease  **Patient Type:** Physiological Diagnosis of AUD | **Race/Ethnicity:**  **N/R**  **Age**: 46.8±10.3  **BMI/Weight:** NR | **Diet tool used**: diet history interview  **Timeframe of assessment**: 6 month recall  **Diet assessment conductor**: dietician  **Diet Quality type**: NR | **Energy including alcohol:** NR  **Energy excluding alcohol:** 2834.7 kcal/day  **Alcohol (g):**148.9±15.1 | **AUD:**  324.5 ±30.3 g/day | **AUD:**  95.9 ±6.5 g/day | **AUD:**  127.5 ±10.6  g/day | Between participants with cirrhosis and participants with pancreatitis, alcohol and nutritional intake did not differ, and both groups were described as well nourished. |
| **Reference** | **Study sample information** | **Study design and history of alcohol use** | **Demographics** | **Dietary assessment information** | **Energy and alcohol intake** | **Macronutrients**  **Carbohydrate Protein Fat** | | | **Summary and dietary intake findings**  Were participants taking in normal amount of food and macronutrients? |
| Yang et al., 2022 | **Dietary Period of Assessment during Study:** Inpatient  **AUD Population Sample Size:** Total: 22 M: 14 F: 8  **Diet as Primary Outcome:** Yes | **Study Design:** Observational  **Drinking status**: Abstinent  **Diagnosis Type:**  AUD (DSM-5)  **Patient Type:** Clinical Diagnosis of AUD | **Race/Ethnicity:**  White, African American, Multiracial, Unknown  **Age**: 46.3±13  **BMI**:23.9±2.5 | **Diet tool used**: food record/diary  **Timeframe of assessment**: Diet history  **Diet assessment conductor**: Nutritionist  **Diet Quality type**: HEI | **Energy including alcohol:** 2665 kcal/day  **Energy excluding alcohol:** 2665 kcal/day  **Alcohol (g):** NR | **AUD:**  45.9% | **AUD:**  19.1% | **AUD:**  34.9% | Female participants were found to consume enough energy to meet their estimated needs, while male participants were found to consume more energy than they needed. |
| Yung & Gordis, 1982 | **Dietary Period of Assessment during Study:** Real-world (half-way house)  **Sample Size:** Total: 70 M: 59 F: 11  **Diet as Primary Outcome:** Yes | **Study Design:** Observational  **Drinking status**: Abstinence/recovery   **Diagnosis Type:**  Alcohol dependence (not specified)  **Patient Type:** Clinical Diagnosis of AUD | **Race/Ethnicity:**  N/R  **Age**:41±10  **Weight:** 74.0±13.4 kg | **Diet tool used**: diet recall  **Timeframe of assessment**: 24 hr recall  **Diet assessment conductor**: Nutritionist  **Diet Quality type**: RDA | **Energy including alcohol:** 37±12 kcal/kg/day  **Energy excluding alcohol:** NR  **Alcohol (g):**NR | **AUD:**  45% | **AUD:**  13% | **AUD:**  42% | Participants met or exceeded RDA on all nutrients except 4 micronutrients- vitamin B, Magnesium, Iron, and Zinc |

**Legend**: Full extraction data table with all included articles. Abbreviations: not reported (N/R); alcohol use disorder (AUD); Female (F); Male (M); Body Mass Index (BMI); Recommended dietary allowance (RDA); Healthy Eating Index-2015(HEI); Diagnostic and Statistical Manual of Mental Disorders (DSM); Comorbidity Alcohol Risk Evaluation Tool (CARET); Missouri Alcohol Severity Scale (MASS); Severity of Alcohol Dependence Questionnaire (SADQ); Alcohol Use Disorders Identification Test (AUDIT); Munich- Composite International Diagnostic Interview (M-CIDL); Alcohol- associated Liver Disease (ALD)

**Supplemental Table S2: Data Extraction Template**

| General study information | \| Author, Year Published \| \| --- \| \| Paper # \| \| Full Citation \| \| DOI \| \| Title \| \| Journal paper was published in \| \| Data Collection Location \| |
| --- | --- | --- | --- | --- | --- | --- | --- | --- |
| Sample demographics/ population descriptors | \| Sample Size \| \| --- \| \| Race/Ethnicity \| \| BMI/Weight (Mean or Median) \| \| Male Participants (N or %) \| \| Female Participants (N or %) \| \| Age in Years (Mean/Median) \| \| Inpatient/Outpatient \| \| Alcohol associated diagnosis (AUD, ALD, Alcoholic cirrhosis, etc.) \| |
| Dietary assessment metrics | \| Dietary Assessment Methodology Tool \| \| --- \| \| Dietary Assessment Conductor \| \| Year dietary assessment was performed \| \| Collection Methodology Limitation Notes \| \| Participant Drinking Status during Dietary Collection \| \| Diet Quality Assessment Metric \| \| Was Nutritional Status measured in the paper? How? \| \| Reported Dietary Information? (Y/N) \| \| Dietary Intake Table? \| \| Research Aim(s) Description \| |
| Dietary intake metrics | \| Total Kcal/day (Mean/Median) \| \| --- \| \| Excluding alcohol Kcal/day (Mean/Median) \| \| Narrative information regarding overall energy intake \| \| % of energy from carbs (or grams) \| \| % of energy from protein (or grams) \| \| % of energy from fat (or grams) \| \| Fiber intake (g/day) \| \| Narrative information regarding macronutrient intake \| \| Alcohol Intake (Mean/Median) \| \| Information about Alcohol Intake \| \| Narrative information regarding Micronutrients \| \| Other food types reported? \| |
| Other | \| Describe Study Design \| \| --- \| \| Research Aim(s) Description \| \| Main Finding Notes \| |

**Legend:** Template used for full data extraction

**Supplementary Table S3: Data Conversions for Articles**

| **Reference** | **Data Conversions** |
| --- | --- |
| Addolorato et al., 1997 | None |
| Addolorato, et al., 1998 | - ***Energy including alcohol:*** 13460 kJ/day * 1kcal/4.184 kJ = **3217.02 kcal/day** - ***Energy excluding alcohol:*** 7690 kJ/day*1 kcal/4.184 kJ = **1837.95 kcal/day** - ***Alcohol:***195g Alc /day * 7kcal/g of alcohol =**1365 kcal alcohol** |
| Addolorato et al., 2000 | None |
| Amadieu et al., 2021 | - ***Alcohol:*** 132 g alcohol /day * 7kcal/g of alcohol =**924 kcal alcohol** |
| Bergheim et al., 2003 | There were 3 AUD populations in this study: ALD I, II, III (I and II were non cirrhotic, III was cirrhotic), so all averages and work were averages and kJ/day to kcal/day calculations were done for the energy including alcohol and energy excluding alcohol   - ***AUD Population Sample Size:*** ALD I= 32 people, ALD II =25 people, ALD III= 19 people, 32+25+19 =**76 people, all males** **mentioned in study** - ***Age:*** ALD I= 45, ALD II =42, ALD III= 54, ((45*32) +(42*25) +(54*19))/76 = **46.26 years**; SE: ((11*32) +(9*25) +(11*19))/76= **10.34 years** - ***BMI:*** ALD I= 24.1, ALD II =24.2, ALD III= 24.7, ((24.1*32) +(24.2*25) +(24.7*19))/76 = **24.28 kg/m^2**; SE: ((5*32) +(5*25) +(3*19))/76 =**4.50 kg/m^2** - ***Energy including alcohol:*** ALD I= 14042, ALD II =14231, ALD III= 14588, ((14042*32) +(14231*25) +(14588*19))/76 = **14240.67 kJ/day**; SE: ((4977*32) +(4305*25) +(3965*19))/76 = **4502.95 kJ/day**; 14240.67 kJ/day * 1kcal/4.184 kJ = **3403.60 kcal/day** - ***Energy excluding alcohol:*** ALD I= 9615, ALD II =10086, ALD III= 10455, ((9615*32) +(10086*25) +(10455*19))/76 = **9979.93 kJ/day**; SE: ((3588*32) +(3227*25) +(2448*19))/76 = **3184.25 kJ/day**; 9979.93 kJ/day * 1kcal/4.184 kJ = **2385.26 kcal/day** - ***Alcohol (g):*** ALD I= 142, ALD II =155, ALD III= 129, ((142*32) +(155*25) +(129*19))/76 = **143.03 g/day**; SE: ((110*32) +(74*25) +(72*19))/76 = **88.66 g/day**; 143.03 g alcohol /day * 7kcal/g of alcohol =**1001.21 kcal alcohol** - ***AUD Carb:*** ALD I= 57, ALD II =56, ALD III= 55, ((57*32) +(56*25) +(55*19))/76 **= 56.17%**; SE: ((9*32) +(12*25) +(8*19))/76 = **9.74%** - ***AUD Protein:*** ALD I=13, ALD II =14, ALD III= 13, ((13*32) +(14*25) +(13*19))/76 **= 13.33%**; SE: ((3*32) +(3*25) +(2*19))/76 =**2.75%** - ***AUD Fat:*** ALD I= 29, ALD II =30, ALD III= 31, ((29*32) +(30*25) +(31*19))/76 **= 29.83%**; SE: ((7*32) +(10*25) +(7*19))/76 = **7.99%** |
| Campillo et al., 2001 | Male (24) and Female (13) were separate, so everything below is an average of those two groups for the patients with cirrhosis (they had controls and healthy controls, but controls had Wernicke’s and healthy controls not added into the calculations)   - ***Age:*** Male= 51.7, Female = 56.1, ((51.7*24)+(56.1*13))/37 = **53.3 years**; SE: ((10.6*24)+(7.4*13))/37= **9.5 years** - ***BMI:*** ((22.3*24)+(22.6*13))/37 = **22.4 kg/m^2**; SE: ((4.0*24)+(4.4*13))/37 = **4.1 kg/m^2** - ***Energy including alcohol:*** ((2078*24)+(1637*13))/37 = **1923 kcal/day**; SE: ((389*24)+(369*13))/37 =**382 kcal/day** |
| Chang et al., 2003 | None |
| Cunha et al., 2004 | None |
| De Timary et al., 2012 | - ***Energy including alcohol:*** 39.0 kcal/kg/day *73.7 kg (this was found through the weight given for the average of female and males) = **2874.3 kcal/day** - ***Energy excluding alcohol:*** 23.5 kcal/kg/day *73.7 kg (this was found through the weight given for the average of female and males) = **1731.95 kcal/day;** 159.4 g alcohol /day * 7kcal/g of alcohol =**1115.8 kcal alcohol** |
| Delarue et al., 1996 | - ***Energy including alcohol:*** 10.0 MJ/day *239.005736 kcal/1MJ = **2390.06 kcal/day;** 185g alcohol /day * 7kcal/g of alcohol =**1295 kcal alcohol** |
| Gloria et al., 1997 | Calculations not convertible because no weight in kg was provided for the units |
| Hurt et al., 1981 | Alcohol is in three different times prior to admission for total AUD population which is an average   - ***Alcohol:*** ((201*58)+(149*58)+(133*58))/(58*3)=**161 g**; SE: ((97*58)+(97*58)+(93*58))/(58*3)**= 95.67 g;** 161g Alc /day * 7kcal/g of alcohol =**1127 kcal alcohol** |
| Laitinen et al., 1990 | - ***Alcohol:*** 131g Alc /day * 7kcal/g of alcohol =**917 kcal alcohol** |
| Lévy et al., 1995 | This was an average of ACP and AC populations and then that is how the kg were averaged and then 1 week is 7 days for the kcal/day conversion   - ***Population Size:*** (ACP=56, AC= 50), 56+50 =**106 people (all Male specified)** - ***Age:*** ((44.8*56)+(49.2*50))/106= **46.9 yr**; SE: ((8.4*56)+(11.0*50))/106 = **9.6 yr** - ***Weight:*** ((67.4*56)+(70.2*50))/106= **68.7 kg**; SE: ((11.8*56)+(11.1*50))/106 = **11.5 kg** - ***Energy including alcohol:*** ((434*56)+(384*50))/106= **410.4 kcal/kg/wk ;** 410.4 kcal/kg/wk*68.7 kg/7days/1wk = **4027.9 kcal/day** - ***Energy excluding alcohol:*** ((331*56) +(261*50))/106= **298 kcal/kg/wk ;** 298 kcal/kg/wk*68.7 kg/7days/1wk =**2924.5 kcal/day** - ***Alcohol:*** ((23.6*56)+(32.1*50))/106= **27.6%** - ***AUD Carbs:*** ((29.1*56)+(29.5*50))/106= **29.3%** - ***AUD Protein:*** ((11.7*56)+(10.1*50))/106= **11%** - ***AUD Fat:*** ((35.7*56)+(28.3*50))/106= **32.2%** |
| Manari et al., 2003 | - ***Energy including alcohol:*** 12300 kJ/day *1kcal/4.184 kJ=**2939.77 kcal/day** - ***Energy excluding alcohol:*** 6030 kJ/day *1kcal/4.184 kJ=**1441.20 kcal/day;** 162g alcohol /day * 7kcal/g of alcohol =**1134 kcal alcohol** |
| Manocha et al., 1989 | - ***Alcohol*** (told they were found to be consuming over 130 grams of alcohol per day, so did 131 to account for it): 131g alcohol /day * 7kcal/g of alcohol =**917 kcal alcohol** |
| Melgaard et al., 1989 | - ***Energy including alcohol:*** 8670 kJ/day *1kcal/4.184 kJ= **2072.18 kcal/day** |
| C. Mendenhall et al., 1985 | Both control and nutrition therapy groups are AUD populations, so there are averages for all of the points and they had Day 0 (pre-hospitalization) and Day 30 (after hospitalization), so we used Day 0 data for the averages   - ***AUD Population:*** (34 +18 (this 18 came from paper not the table because they explain that initially there were 23 but 5 were excluded, so that is 18, not 17 ( 17 only for the age was used since the table had 18 for the other measurements) = **52 people ( M/F not written in paper)** - ***Age:*** ((49.3*34)+(44.7*17))/51= **47.8 years**; SE: ((1.6*34)+(2.1*17))/51= **1.8 years** - ***Energy of Alcohol:*** ((1840*34)+(1640*18))/52 =**1770.8 kcal/day;** SE: ((204*34)+(282*18))/52 =**231 kcal/day** - ***Energy including Alcohol:*** ((3604*34)+(3080*18))/52 = **3422.6 kcal/day;** SE: ((255*34)+(353*18))/52 = **288.9 kcal/day** - ***Energy excluding Alcohol:*** 3422.6-1770.8= **1651.8 kcal/day**; SE: 288.9-231 = **57.9 kcal/day** - ***AUD Protein:*** ((56*34) +(53*18))/52 =**55 g/day**; SE: ((5*34)+(6*18))/52 = **5.4 g/day** - ***Alcohol:*** ((265.5*34) +(236.6*18))/52 = **255.5 g/day**; SE: ((29.5*34) +(40.7*18))/52 = **33.4 g/day** |
| C. L. Mendenhall et al., 1993 | Both active treatment and placebo groups are AUD populations, so there are averages for all the points   - ***AUD Population:*** (137+136) = **273 people (all M)** - ***Age:*** ((50*137) +(51*136))/271 = **50.9 years**; SE: ((10*137) +(9*136))/271 = **9.6 years** - ***Energy including alcohol:*** ((2830*137) +(2637*136))/271 = **2754 kcal/day**; SE: ((1573*137) +(1417*136))/271 = **1506.3 kcal/day** - ***Alcohol:*** ((221*137) +(188*136))/271= **206.1 g/day**; SE: ((170*137) +(180*136))/271= **176.3 g/day**; 206.1 g alcohol /day * 7kcal/g of alcohol =**1442.7 kcal alcohol** |
| Mezey et al., 1988 | AUD population was divided into pancreatitis, moderate/severe alcohol associated hepatitis, and cirrhosis (all AUD based checked within paper) so researchers averaged the numbers for all the ratios   - ***AUD Population Size***: 42+50+19+18 = **129 people**; Male: 25+32+15+8 = **80 people**; Female: 17+18+4+10 = **49 people** - ***Age***: ((40.7*42)+(41.8*50)+(42.9*19)+(47.7*18))/129 = **42.4 years**; SE: ((1.6*42)+(1.5*50)+(2.2*19)+(2.2*18))/129 = **1.7 years** - ***Weight (% of standard):*** ((92.4*42)+(103*50)+(100.4*19)+(104.4*18))/129 = **99.4%**; SE :( (2.6*42)+(2.9*50)+(5.0*19)+(4.1*18)/129 = **3.3%** - ***Energy including alcohol:*** ((2514*42)+(2627*50)+(2203*19)+(2572*18))/129 =**2520.1 kcal/day**; SE: ((142*42)+(113*50)+(134*19)+(151*18))/129 = **130.8 kcal/day** - ***Energy excluding alcohol:*** ((1269*42)+(1276*50)+(1075*19)+(1364*18))/129 =**1256.4 kcal/day**; SE: ((97*42)+(80*50)+(83*19)+(151*18))/129= **95.9 kcal/day** - ***Alcohol Intake:*** ((177.8*42)+(191.6*50)+(165.8*19)+(178.1*18))/129 = **181.4 g/day;** SE: ((14.5*42)+(12.3*50)+(17.8*19)+(23.4*18))/129 = **15.4 g/day**; 181.4g alcohol /day * 7kcal/g of alcohol =**1269.8 kcal alcohol** - ***AUD protein (%)*** : ((9.1*42)+(8.2*50)+(7.1*19)+(8.8*18))/129 =**8.4%**; SE: ((0.6*42)+(0.4*50)+(0.5*19)+(1.4*18))/129= **0.6%** - ***AUD Carbs (%):*** ((22.1*42)+(21.0*50)+(20.6*19)+(23.0*18))/129 =**21.6%**; SE: ((1.3*42) +(1.1*50)+(2.3*19)+(2.4*18))/129 = **1.5%** - ***AUD Fat (%):*** ((19.3*42) +(19.8*50) +(20.4*19)+(21.2*18))/129 =**19.9%**; SE: ((1.3*42) +(1.2*50)+(1.9*19)+(2.6*18))/129 = **1.5%** |
| Mills et al., 1983 | No other population mentioned other than AUD pop; calculated alcohol calories by calculating the difference between kcal/day - kcal/day (excluding alcohol)   - Alcohol intake calories were 52% on average from the total caloric intake, **2452*.52=1275.04 kcal/day** |
| Neville et al., 1968 | Age and weight were reported individually for each of the 34 participants, so it had to be averaged by researchers.   - ***Age (yrs):*** (25+26+29+35+37+37+37+37+38+38 +40+40+41+44+45+47+48+49+53+54 +55+55+55+56+56+59+25+26+34+38 +42+43+47+48)/34 = **42.32 years** - ***Weight (lbs) :(***154+144+152+184+188+167+130+111+151+156 +145+148+174+181+132+179+154+96+162+142 +128+154+152+116+113+135+188+128+107+130 +100+88+126+117)/34 = **142.12 lbs**   Energy and Macronutrients was reported for men (n=26) and women (n=8) separately and had to be averaged by researchers.   - ***Energy including alcohol:*** ((2710*26) +(2578*8))/34 = **2678.94 kcal/day**; SE: ((933*26) +(1459*8))/34= **1056.76 kcal/day** - ***AUD Carbs (%):*** ((36.4*26) +(22*8))/34 = **33.0%** - ***AUD Protein (%):*** ((10*26) +(11.2*8))/34 = **10.3%** - ***AUD Fat (%):*** ((22.6*26) +(27.6*8))/34 = **23.8%** - ***Alcohol (g):*** ((141*26) +(81*8))/34 = **126.9 g/day;** SE: ((97*26) + (72*8))/34 = **91.12 g/day**; 126.9 g alcohol /day * 7kcal/g of alcohol =**888.3 kcal alcohol** |
| NICOLÁS et al., 1993 | - ***Alcohol:*** 235g Alc /day * 7kcal/g of alcohol =**1645 kcal alcohol** |
| Nielsen et al., 1993 | - ***Energy including alcohol***: 7.2 MJ/day*239.005736 kcal/1MJ = **1720.84 kcal/day** |
| NOEL-JORAND & BRAS, 1994 | Age was reported separately rather than having a GR2 value and had to be averaged by researchers through the GR3 and GR4 groups. GR2 was separated further into GR3 and GR4 groups, but GR2 was the AUD group so that was used in all the supplemental doc, no SE in this paper other than Age and BMI; GR#= Group Number   - ***AUD population size*** (it was said that the alcohol group GR2 and GR1, the control group had the same percentage of M to F, which was 18% Female, so that was used for the calculations for the GR2 group): 72*.18 = **13 female;** 72-13 female = **59 male** - ***Age (yrs):*** ((39.6*31) +(48*41))/72 = **44.38 years**; SE: ((6*31) +(9.7*41))/72 = **8.11 years** - ***Energy including alcohol*** (this one had the energy for alcohol separate so added them to find the total): 2842+1534= **4376 kcal/day** - ***AUD Carb*** (this was the only one of the AUD macronutrients that had the carbs separated for alcohol and nonalcohol, so they were added to find the total): 371.3+49.7= **421 g/day** |
| Palliyath & Schwartz, 1993 | - ***Alcohol:*** 207g alcohol /day * 7kcal/g of alcohol =**1449 kcal alcohol** |
| Panagaria et al., 2007 | The AUD population was split into two groups (ALD (n=41) and AUD (n=25)) and had to be averaged by researchers.   - ***AUD Population Size:*** 41+25= **66 people** (all male specified in the paper) - ***Age (yrs):*** ((36.28*25) +(42.51*41))/66 = **40.15 years;** SE: ((6.01*25) +(7.54*41))/66 = **6.96 years** - ***BMI:*** ((21.08*25) +(22.30*41))/66 = **21.84 kg/m^2**; SE: ((2.63*25) +(3.49*41))/66 = **3.16 kg/m^2** - ***Energy including alcohol:*** ((1006.42*25) +(969.78*41))/66 = **983.66 kcal/day**; SE: ((371.83*25) +(278.08*41))/66 = **313.59 kcal/day** - ***AUD Carbs (g/day):*** ((169.15*25) +(178.05*41))/66 = **174.68 g/day**; SE: ((80.97 *25) +(88.57*41))/66 = **85.69 g/day** - ***AUD Protein (g/day):*** ((42.08*25) +(36.31*41))/66 = **38.5 g/day**; SE: ((24.01*25) +(21.30*41))/66 = **22.33 g/day** - ***AUD Fat (g/day):*** ((21.04*25) +(40.11*41))/66 = **32.89 g/day**; SE: ((18.25*25) +(26.25*41))/66 = **23.22 g/day** - ***Alcohol (g/day):*** ((188.64*25) +(145.17*41))/66 = **161.64 g/day**; SE: ((109.05*25) +(137.75*41))/66 = **126.88 g/day;** 161.64g alcohol /day * 7kcal/g of alcohol =**1131.48 kcal alcohol** |
| Pezzarossa et al., 1986 | Age and Weight were reported individually (also divided into Groups A-C) and had to be averaged by the researchers.   - ***Age (yrs):*** (25+48+42+43+39+36+45+40+48+25 +27+47+38+28+31+45+28+36+44+49 +35+32)/22 = **37.77 years**; SE: ((2*8) + (3*8) + (3*6))/22 = **2.64 years** - ***Weight (kg):*** (80+70+59+47+70+74+66+70+78+67 +59+55+77+65+62+55+62+67+61+63 +76+70)/22 = **66.05 kg**; SE: ((4*8) + (3*8) + (2*6))/22 = **3.09 kg** - ***Energy Excluding Alcohol:*** (1400+1400+1000+750+1400+1600+1200+900+1200+1300+1100+1000+1300+1500+1000+900+900+1200+1400+1000+1300+900)/22 = **1165.91 kcal/day;** SE: ((105*8) + (71*8) + (87*6))/22 = **87.73 kcal/day** - ***Alcohol:***(300+200+250+250+200+250+200+250+300+200+200+200+250+300+200+200+200+250+300+200+250+200)/22 = **234.09 g**; SE: ((13*8) + (16*8) + (17*6))/22 = **15.18 g;** 234.09g alcohol /day * 7kcal/g of alcohol =**1638.63 kcal alcohol** |
| Pitchumoni et al., 1980 | AUD population was split into two groups (cirrhosis and pancreatitis) and had to be averaged by researchers; for the Alcohol intake per day, authors report >200g but changed to 201g for the calculations   - ***Energy excluding alcohol:*** ((810*26)+(1050*36))/62 = **949.35 kcal/day**; SE: ((330*26)+(523*36))/62 = **442.06 kcal/day** - ***AUD Protein (g):*** ((56*26)+(67*36))/62 = **62.4 g/day**; SE: ((23*26)+(22*36))/62 =**22.42 g/day** - ***AUD Fat (g):*** ((40*26)+(59*36))/62 = **51.0 g/day**; SE: ((22*26)+(33*36))/62 = **28.39 g/day** |
| Ratteree et al., 2019 | - ***Energy including alcohol:*** 27961.7 kJ/day*1kcal/4.184 kJ=**6683.01 kcal/day** - ***Energy excluding alcohol:*** 14109.3 kJ/day*1kcal/4.184 kJ=**3372.20 kcal/day** - ***Alcohol: No*** calculations needed, but need to mention we talked to authors of the paper and they shared that it was 472.96 ± 75.8 g, which was incorrectly written in the paper as 4000 |
| Rintamäki et al., 2014 | Populations were divided by Male and Female (AUD one too, so must take that into account)   - ***Age:*** ((45.1*33) +(46.1*167))/200 = **45.94 years**; SE: ((12.0*33) +(10.3*167))/200 = **10.58 years** - ***BMI:*** ((26.6*33) +(27.1*167))/200 = **27.02 kg/m^2;** SE: ((4.8*33) +(4.0*167))/200= **4.13 kg/m^2**   For the energy and macronutrient intake, the population numbers changed, so kept that consistent in supplemental table we have the original numbers since there was inconsistency in the paper   - ***Energy including Alcohol:*** ((8.8*31) +(9.8*150))/181 = **9.63 MJ/day**; 9.63 MJ/day *239.005736 kcal/1MJ = **2301.63 kcal/day** - ***Alcohol:*** ((2.8*31) +(4.5*150))/181 = **4.21%;** 4.21% of 2301.63kcal/day = **96.9 kcal/day** - ***AUD Carbs:*** ((40*31) +(41*150))/181 = **40.83%** - ***AUD Protein:*** ((18*31) +(17*150))/181 = **17.17%** - ***AUD Fat:*** ((39*31) +(37*150))/181 = **37.34%** |
| Sangwan & Khetarpaul, 1998 | None |
| Sarin et al., 1997 | AUD population was split into two groups (Group 1 = ALD, and Group 2 = Chronic AUD) and had to be averaged by researchers   - ***Population Size:*** 67+52 = **119 people (not specified male or female)** - ***Age (yrs):*** ((43.1*67) +(37.9*52))/119 = **40.8 years**; SE: ((8.7*67) +(8.9*52))/119 = **8.79 years** - ***Weight (kg):*** ((61.9*67) +(58.2*52))/119 = **60.3 kg**; SE: ((11*67) +(10*52))/119 = **10.56 kg** - ***Energy including alcohol*** (this one had the energy for alcohol separate so added them to find the total): Alcoholic calories: ((1299*67) +(1423*52))/119 = **1353.19 kcal/day;** SE: ((1016*67) +(1031*52))/119 = **1022.56 kcal/day**; Total Calories: 1353.19+ 1481.45 = **2834.64 kcal/day**: SE: (1022.56+ 493.56)/2 = **758.06 kcal/day** - ***Energy excluding alcohol:*** ((1547*67) +(1397*52))/119 = **1481.45 kcal/day**; SE: ((494*67) +(493*52))/119 =**493.56 kcal/day** - ***AUD Carbs (g):*** ((214*67) +(119*52))/119 = **172.5 g/day**; SE: ((75*67) +(75*52))/119 =**75 g/day** - ***Protein (g):*** ((50.4*67) +(47.3*52))/119 = **49 g/day**; SE: ((22*67) +(18.4*52))/119 = **20.43 g/day** - ***Fat (g):*** ((49.3*67) +(45*52))/119 = **47.4 g/day;** SE: ((19*67) +(15.5*52))/119 = **17.47 g/day** - ***Alcohol (g):*** ((187*67) +(194*52))/119 = **190.1 g/day**; SE: ((150*67) +(115*52))/119 = **134.71 g/day;** 190.1g alcohol /day * 7kcal/g of alcohol =**1330.7 kcal alcohol** |
| Simko et al., 1982 | AUD divided between A and B (A= AUD people with liver disease, B= AUD people with no liver disease) so averaged then used for calculations   - ***Population Size:*** 62+20=**82 people**; Male: 57+18= **75**; Female: 82-75 = **8** - ***Age:*** ((49.9*62) +(55.7*20))/82 = **51.3 years;** SE: ((1.4*62) +(2.3*20))/82 =**1.62 years** - ***Weight:*** ((72*62) +(67.3*20))/82 = **70.9 kg**; SE: ((2.1*62) +(2.5*20))/82 = **2.20 kg** - ***Energy including alcohol:*** ((28.1*62) +(35.3*20))/82 = **29.86 kcal/kg/day** *70.9 kg (as found from the previous calculation) = **2116.80 kcal/day**; SE (no need for further calculations): ((1.8*62) +(2.8*20))/82= **2.04 kcal/kg/day** - ***Alcohol:***( (2.05*62) +(1.80*20))/82 = **1.99 g/kg/day** *70.9 kg (as found from the previous calculation) = **141.02 g/day**; SE (no need for further calculations): ((0.15*62) +(0.22*20))/82= **0.17 g/kg/day**; 141.02g alcohol /day * 7kcal/g of alcohol =**987.14 kcal alcohol** - ***AUD Protein:*** ((0.9*62) +(1.11*20))/82 = **0.95 g/kg/day** *70.9 kg (as found from the previous calculation) = **67.44 g/day**; SE (no need for further calculations): ((0.05*62) +(0.08*20))/82= **0.06 g/kg/day** |
| Sobral-Oliveira et al., 2011 | AUD population was split into two groups (chronic alcohol-associated pancreatitis is Group 1 and chronic AUD is group 2) and averaged by researchers   - ***Population size:*** 20+12 =**32 people (all Male)** - ***Age (yrs.):*** ((54.1*20) +(55.1*12))/32 = **54.5 years**; SE: ((11.4*20) +(8.5*12))/32 =**10.3 years** - ***BMI:*** ((23.7*20) +(26.2*12))/32 = **24.6 kg/m^2**; SE: ((4.6*20) +(6.8*12))/32 = **5.4 kg/m^2** - ***Energy excluding alcohol:*** ((1637*20) +(1645*12))/32 = **1640 kcal/day**; SE: ((546*20) +(310*12))/32 = **457.5 kcal/day** - ***AUD Carb:*** ((224*20) +(244*12))/32 = **231.5 g/day**; SE: ((87*20) +(50*12))/32 =**73.1 g/day** - ***AUD Protein:*** ((71*20) +(62*12))/32 = **67.6 g/day**; SE: ((24*20) +(14*12))/32 = **20.3 g/day** - ***AUD Fat:*** ((51*20) +(48*12))/32 = **49.9 g/day**; SE: ((17*20) +(14*12))/32 = **15.9 g/day** - ***Alcohol:*** ((252.5*20) +(408.4*12))/32 = **311.0 g/day**; SE: ((229.1*20) +(187.4*12))/32 = **213.5 g/day;** 311g alcohol /day * 7kcal/g of alcohol = **2177 kcal alcohol** |
| Videla et al., 1984 | The AUD population is divided into two, patients with liver necrosis and patients without liver necrosis so must average; While it is stated in the paper that 45 patients were studied, only 27 of them provided a weight measurement, and 24 of them provided dietary intake, hence why these numbers are divided from those values   - ***Weight (% of ideal):*** ((104.8*10) +(96.5*17))/27 = **99.6 %**; SE: ((5.1*10) +(2.8*17))/27 = **3.7%** - ***Energy excluding alcohol (ideal weight/kcal*kg):*** ((14.9*9) +(13.2*15))/24 = **13.8 ideal weight/kcal/kg**; SE: ((5.1*9) +(3.0*15))/24 = **3.8 ideal weight/kcal/kg** - ***Energy of alcohol*** (this one had the energy for alcohol separate so added them to find the total): ((28.7*9) +(35.4*15))/24 =**32.9 ideal weight/kcal/kg;** SE: ((3.6*9) +(3.6*15))/24 = **3.6 ideal weight/kcal/kg** - ***Energy Including alcohol:*** 13.8+32.9= **46.7 ideal weight/kcal/kg**; SE: (3.8+3.6)/2 = **3.7 ideal weight/kcal/kg** - ***AUD Protein (ideal weight/g*kg):*** ((0.63*9) +(0.44*15))/24 = **0.51 ideal weight/g/kg**; SE: ((0.20*9) +(0.10*15))/24 = **0.14 ideal weight/g/kg** |
| Wagnerberger et al., 2007 | The AUD population was split into 6 groups (ALD 1, ALD 2, and ALD 3, all of which were divided further into male and female, calculations are ALD# M, ALD#F added) and was averaged by researchers   - ***Population Sample Size:*** Male: 82+45+31 = **158 people**; Female: 16+14+22 =**52 people**; Total: 158+52 = **210 people** - ***Age (yrs):*** ((45*82) +(50*16) +(45*45) +(42*14) +(51*31) +(48*22))/210 **= 46.4 years;** SE: ((1.0*82) +(1.5*16) +(1.5*45) +(2.8*14) +(1.4*31) +(1.8*22))/210= **1.4 years** - ***BMI:*** ((25*82) +(23*16) +(24*45) +(20*14) +(26*31) +(24*22))/210 = **24.3 kg/m^2**; SE: ((0.5*82) +(0.7*16) +(0.7*45) +(0.8*14) +(1.0*31) +(1.1*22))/210 =**0.7 kg/m^2** - ***Alcohol (g/day):*** ((142*82) +(79*16) +(151*45) +(104*14) +(123*31) +(87*22))/210 = **128 g/day**; SE:( (12.6*82) +(7.3*16) +(12.6*45) +(15.0*14) +(13.0*31) +(8.9*22))/210= **12.0 g/day;** 128g alcohol/day * 7kcal/g of alcohol = **896 kcal alcohol** - ***Energy including alcohol:*** ((3812*82) +(2789*16) +(3413*45) +(2500*14) +(3215*31) +(2274*22))/210 = **3311.8 kcal/day**; SE: ((189*82) +(116*16) +(188*45) +(255*14) +(173*31) +(189*22))/210 =**185.3 kcal/day** - ***Energy excluding alcohol: (***(2807*82) +(2230*16) +(2337*45) +(1760*14) +(2340*31) +(1654*22))/210 = **2402.8 kcal/day**; SE:( (153*82) +(112*16) +(145*45) +(216*14) +(118*31)+(169*22))/210 = **148.9 kcal/day** - ***AUD Protein (g):***( (101*82) +(85*16) +(83*45) +(62*14) +(85*31) +(58*22))/210 = **86.5 g/day**: SE: ((5.7*82) +(7.1*16) +(5.4*45) +(9.6*14) +(5.4*31) +(6.5*22))/210 =**6.0 g/day** - ***AUD Fat (g):*** ((111*82) +(95*16) +(85*45) +(70*14) +(86*31) +(69*22))/210 = **93.4 g/day**; SE: ((6.7 *82) +(7.7*16) +(7.7*45) +(10.5*14) +(4.5*31) +(8.7*22))/210 = **7.1 g/day** - ***AUD Carbs (g):*** ((335*82) +(238*16) +(285*45) +(187*14) +(288*31) +(214*22))/210 = **287.4 g/day**; SE:( (19.6*82) +(21.6*16) +(18.7*45) +(27.9*14) +(17.5*31) +(24.4*22))/210 =**20.3 g/day** |
| Wilkens Knudsen et al., 2014 | - ***Energy including alcohol:*** 8401 kJ/day*1kcal/4.184 kJ = **2007.89 kcal/day**; 171g Alc /day * 7kcal/g of alcohol =**1197 kcal alcohol** |
| Wilson et al., 1985 | The AUD population was divided into 2 populations, alcohol-associated pancreatitis, alcohol-associated cirrhosis, so averaged it and then calculated the conversions   - ***Population Size:*** 20+33 = **53 people *(M/F not specified in paper, they gave a ratio, but the ratio was not decipherable for calculations)*** - ***Age (yrs):*** ((40*20) +(51*33))/53 = **46.8 years**; SE: ((9*20) +(11*33))/53= **10.3 years** - ***AUD Protein (g):*** ((109*20) +(88*33))/53 = **95.9 g/day**; SE: ((9*20) +(5*33))/53 = **6.5 g/day** - ***AUD Fat (g):*** ((148*20) +(115*33))/53 = **127.5 g/day**; SE: ((15*20) +(8*33))/53 = **10.6 g/day** - ***AUD Carbs (g):*** ((383*20) +(289*33))/53 = **324.5 g/day**; SE: ((39*20) +(25*33))/53 =**30.3 g/day** - ***Alcohol (g):*** ((147*20) +(150*33))/53 = **148.9 g/day**; SE: ((17*20) +(14*33))/53 = **15.1 g/day;** 148.9 g alcohol /day * 7kcal/g of alcohol = **1042.3 kcal alcohol** - ***Energy excluding alcohol:*** (13856*20+10651*33)/53 = 11860.4 kJ/day*1kcal/4.184 kJ =**2834.7 kcal/day**; SE: (1289*20+731*33)/53 = **941.6 kJ/day *(no need to convert for data analysis, just for supp. Table)*** |
| Yang et al., 2022 | None |
| Yung & Gordis, 1982 | - ***Energy including alcohol***: 37 kcal/kg/day * 74 kg (as found in the tables) = **2738 kcal/day** |

Legend: Tracking of all data conversions for the meta-analysis for any articles that required a conversion. Note: Calculations for the SE/ SDs were not performed unless there were multiple populations, and the averages were mentioned.

**Supplemental Table S4:** **Abbreviations of the diagnostic tools used to determine alcohol misuse across all studies**

| **Diagnosis Type** | **Abbreviation/ Term** | **Definition** |
| --- | --- | --- |
| Physiological diagnosis | ALD or SLD | Alcohol-associated Liver Disease: This is diagnosed primarily through clinical evaluation, liver biopsies, bloodwork, and imaging tests and populations with ALD either had or have alcohol misuse (often AUD)  Steatosis/Severe Liver Disease: Steatosis and severe liver disease, though part of the alcohol-associated liver disease (ALD) spectrum, were coded or labeled as separate diagnoses related to alcohol use in some studies. While alcohol use can also affect the pancreas (e.g., pancreatitis), this is a distinct clinical entity from ALD |
| Clinical Diagnosis | DSM (DSM-5, M-CIDL) | Diagnostic and Statistical Manual of Mental Disorders: This is a primary source of diagnosis for AUD in the US and other countries.  Munich-Composite International Diagnostic Interview: This test is a self-diagnostic interview that is designed for assessing mental disorders according to the definitions of DSM-IV and ICD-10. |
| Questionnaire Diagnosis | MASS | Missouri Alcohol Severity Scale: This is a tool that correlates scores and symptoms data from people with alcohol dependence who are treated in public programs in Missouri. The study this is used in is uses a modified version of this test. |
|  | SADQ | Severity of Alcohol Dependence Questionnaire: This is a diagnosis tool used in the UK. |
|  | AUDIT | Alcohol Use Disorders Identification Test: This 10- question test is used as a method of screening |
|  | CARET | Comorbidity Alcohol Risk Evaluation Tool: This test is used as a diagnosis for AUD primarily in New Zealand and Australia. |

**Legend**: Abbreviation list for diagnostic criteria across all studies.

**Supplemental Table S5:** **Diet Assessment Tool Use by Drinking Status**

| **Diet Assessment Tool Name** | **Abstinent/**  **Inpatient  (N=4)** | **Abstinent/**  **Real-World (N=1)** | **Active Drinking/**  **Real-World  (N=36)** |
| --- | --- | --- | --- |
| Diet History Interview | 0 | 0 | 18 |
| Diet Recall | 2 | 0 | 7 |
| Food Record or Diary | 2 | 1 | 3 |
| Food Frequency Questionnaire | 0 | 0 | 3 |
| Retrospective Structured Questionnaire | 0 | 0 | 2 |
| Unclear | 0 | 0 | 2 |
| Diet Recall and Food Record or Diary | 0 | 0 | 1 |

**Legend**: Different diet assessment tools used outlined by abstinent/inpatient group, abstinent living in the real world group or in the active drinking/real world group. Most dietary intake assessments were conducted for the active drinking real-world group.
